# Navigating Across Heritage and Destination Cultures: How Personal Identity and Social Identification Processes Relate to Domain-Specific Acculturation Orientations in Adolescence

**DOI:** 10.1007/s10964-023-01870-y

**Published:** 2023-09-29

**Authors:** Elisabetta Crocetti, Savaş Karataş, Susan Branje, Beatrice Bobba, Monica Rubini

**Affiliations:** 1 Department of Psychology, Alma Mater Studiorum University of Bologna, Bologna, Italy; 2 Department of Education and Pedagogy, Educational Psychology—Socialisation and Culture Research Group, Martin Luther University Halle-Wittenberg, Halle (Saale), Germany; 3 Department of Youth and Family, Utrecht University, Utrecht, The Netherlands

**Keywords:** Personal identity, Social identification, Acculturation, Education, Friendships, Longitudinal

## Abstract

Personal identity and social identification processes can be challenging for adolescents belonging to an ethnic minority, who have to cope with the acculturation task of navigating several (and often conflictual) alternatives put forth by their cultural heritage community and destination society. Because identity and acculturation tasks are embedded in core domains of adolescents’ life, this three-wave longitudinal study with ethnic minority adolescents (*N* = 244, 43.4% male; *M*_age_ = 14.9) examined how personal identity processes and social identifications are related to acculturation orientations in the education and friendship domains. Results of traditional cross-lagged models showed that, in the educational domain, adolescents who scored higher on cultural heritage maintenance compared to their peers, scored higher on commitment later on. In the friendship domain, stronger associations were found, such that adolescents who scored higher on cultural heritage maintenance compared to their peers, reported higher commitment and in-depth exploration later on, while those who scored higher on identification with friends reported over time also higher cultural heritage maintenance and destination culture adoption. Random-intercept crossed-lagged models indicated that, when adolescents reported above their own average on reconsideration of educational commitment, they reported increased cultural heritage maintenance later on. Furthermore, consistent associations (at baseline and over time) emerged. Overall, this study points to virtuous alliances between the fulfillment of tasks related to adolescents’ identity development and acculturation.

## Introduction

Making important choices in terms of various personal identity domains (e.g., education, friendship) while identifying themselves as members of meaningful social groups can be considered fundamental developmental tasks in adolescence (Crocetti et al., 2018, 2023). Fulfillment of these age-salient identity tasks can be particularly challenging for youth belonging to an ethnic minority, as they have to cope also with the acculturation tasks of negotiating their orientations toward heritage and destination cultures (Berry, 1997; Erentaitė et al., 2018). Identity and acculturation tasks might operate differently in education and friendship domains, in which adolescents can have more or fewer opportunities to explore their own paths (Becht et al., 2016; Ward & Geeraert, 2016). However, despite the differences, these processes are likely to be intertwined, although no prior research has assessed their interplay. For instance, adolescents’ development of their educational identity may be influenced by the maintenance of the cultural norms of the family of origin, which can value education and academic achievement as a main gateway for the social mobility of their ethnic minority children (OECD, 2023). In the friendship domain, how adolescents define their interpersonal identity may reflect the degree to which they maintain their cultural heritage or adopt the destination culture and translate their orientations in the choice of same-ethnic and cross-ethnic friendships (Karataş et al., 2021). In line with this reasoning, this longitudinal study aimed to disentangle how ethnic minority adolescents’ personal identity processes and social identifications are related to acculturation orientations in these core education and friendship domains.

### Personal Identity Processes and Social Identifications in Adolescence as Inextricably Intertwined Processes

Personal identity refers to forming a clear sense of self, including a subjective feeling of continuity across different contexts and times (Branje, 2022; Branje et al., 2021). In Erikson’s (1950, 1968) seminal psychosocial theory, identity formation is conceptualized as the most important developmental task of adolescence. Marcia (1966) further elaborated Erikson’s views by conceptualizing identity statuses as different styles of coping with the identity crisis experienced in adolescence. In this regard, individuals can be classified in distinct identity statuses based on the extent to which they have enacted meaningful commitments in significant life domains, after having explored, or not, different possibilities. Taking a step further, to increase the understanding of the process underlying different identity statuses and the transitions across them, process-oriented models have been proposed (for a review, see Meeus, 2011).

One of these process-oriented models is the three-factor model, which represents a parsimonious approach to understand how individuals form and change their identity over time and embedded in life contexts (Crocetti et al., 2023). This model (Crocetti et al., 2008) taps into the iterative processes of identity development by considering the dynamic interplay between commitment, in-depth exploration, and reconsideration of commitment. *Commitment* refers to enduring choices that adolescents have made about various developmental domains and the self-confidence stemming from these choices. *In-depth exploration* indicates the extent to which adolescents think actively about the commitments they have enacted, reflect on their choices, search for additional information about them, and talk about their commitments with others. *Reconsideration of commitment* refers to comparing current commitments with possible alternatives because the current ones are no longer satisfactory. These three processes form the basis of identity formation and maintenance cycles (Crocetti, 2017). The identity formation cycle builds upon the interplay between being committed and reconsidering current choices in light of other alternatives; while the identity maintenance (or consolidation) cycle relies on exploring in depth available commitments in order to verify and validate them (Meeus, 2018).

These identity processes operate in multiple domains, such as education, friendship, religion, politics, and so on (e.g., Vosylis et al., 2018). In adolescence, the most important domains are education and friendship, which involve questions about which type of education youth desire to pursue in and how they want to be in a relationship with friends (Becht et al., 2016; Branje et al., 2021). In each domain, commitment indicates the youth’s attempts to develop and maintain a coherent sense of self, whereas reconsideration of commitment represents the questioning and re-evaluating the sense of self. In-depth exploration has both positive and negative aspects as it is related to, on the one, curiosity, and, on the other hand, distress and confusion (for a review, see Crocetti, 2017).

Personal identity processes are inextricably intertwined with *social identifications* in adolescence (Albarello et al., 2018; Crocetti et al., 2018). Social identification pertains to the subjective aspects of group memberships and is conceptualized as “the feelings of belonging, affiliation, and connectedness to a group, coupled with the sense of commonality with fellow ingroup members” (Miller et al., 2015, p. 340). As a critical component of social identity (Tajfel & Turner, 1979), social identification encompasses the awareness of the value attributed to group membership, as well as the affective experience related to being a member of a certain group (Ellemers et al., 2002). In this sense, social identification not only represents being part (or member) of a certain social group but also involves the positive attributes of the ingroup and provides essential implications for youth psychosocial adjustment (e.g., social well-being; Albarello et al., 2021).

Individuals can identify with multiple social groups. Some of them are smaller and proximal groups, such as the family and the group of friends, while others are larger and more distal, such as the national and ethnic groups (e.g., Karataş et al., 2023). Notably, a fundamental developmental trajectory of social identification has been documented (Albarello et al., 2021), highlighting that social identification with proximal peer groups poses the basis for identifying later on with more abstract and distant groups, up to identification with humanity. Based on this theoretical grounding, it is of utmost importance considering the role played in adolescence by identification with peer groups.

The two most salient peer groups adolescents can identify with are the groups of their classmates and friends (Albarello et al., 2018). Classmates are not mutually chosen, yet adolescents regularly interact with them under the supervision of teachers. The group of friends gathers peers with whom youth interact voluntarily in their free time across various social settings. Although these two groups may partly overlap, since some adolescents may become friends of their classmates and meet them in their spare out-of-school time, they are characterized by distinct social experiences stemming from the fact that while classmates represent a formal group, friends are an informal one (Albarello et al., 2021). Furthermore, in several educational systems, adolescents choose their secondary school based on the school program. This often results in attending a school that is in a different neighborhood or even municipality. Thus, the group of classmates, met in the school context, may be largely different from the group of friends with whom adolescents interact in their living contexts and in their leisure time.

Associations between social identification with peer groups and identity processes have been theorized, suggesting a dynamic of reciprocal influences (Crocetti et al., 2018), and empirically examined (Albarello et al., 2018). Longitudinal evidence has shown that social identifications with classmates and friends are developmentally related to identity commitment, in-depth exploration, and reconsideration of commitment. More specifically, personal identity processes in the educational and interpersonal domains and social identification with classmates and friends have been found to be associated both concurrently and longitudinally, with most cross-lagged effects showing that social identifications influence personal identity formation and consolidation in the interpersonal identity domain (Albarello et al., 2018). This evidence underscores that symbolic processes involving adolescents as members of meaningful social groups can feed their personal identity development. Thus, personal identity processes and social identifications are inextricably intertwined, leading to the study of “identities” within the lens of a developmental social-psychological perspective (Crocetti et al., 2023).

### Domain-Specific Acculturation Orientations in Adolescence

Acculturation pertains to the psychological and cultural changes that might occur as a result of contact between members of different cultural groups (Berry, 2005). Theorization and research on acculturation have progressively moved away from an initial simplified unidimensional perspective (Gordon, 1964), according to which the acculturation process of individuals from minority groups is a linear process, going from discarding the values, beliefs, attitudes, and behaviors of the cultural heritage to adopting those of the destination culture. In contrast, building upon Berry’s (1997, 2005) bidimensional model, different acculturation strategies (i.e., integration, assimilation, separation, and marginalization), are distinguished based on the degree of maintenance of their cultural heritage and adoption of the destination culture. These two orientations are, thus, conceptualized not as opposite poles of a continuum, but as distinct from each other. Therefore, individuals can find their personal balance (as in the case of integration), prefer one over the other (i.e., assimilation or separation), or show a low endorsement of both (i.e., marginalization).

Taking a step forward, the Relative Extended Acculturation Model (Navas et al., 2005) conceptualized acculturation as a dynamic process in which *cultural heritage maintenance* and *destination culture adoption* processes can operate differently across various life domains. In fact, ethnic minority individuals may easily adopt the values and practices of the destination society in some domains (e.g., adopting the educational norms of the destination society), whereas they may prefer to remain close to their cultural heritage in others (e.g., maintaining their religious beliefs). As for identity, also for acculturation, education and friendships are two core domains (Mancini & Bottura, 2014).

Schools are the pivotal acculturation contexts for youth with diverse migrant backgrounds, such as refugees and first- and second-generation immigrants, coming from different cultural groups and with diverse family backgrounds and histories of migration (Schachner et al., 2018; Suárez-Orozco, 2017). In schools, ethnic minority adolescents, through their interactions with ethnic majority students and teachers can mainly learn about the destination culture (e.g., Vietze et al., 2020). However, when schools support institutional intergroup ideologies in favor of multiculturalism (as opposed to assimilationism), ethnic minority adolescents can also experience a fertile context for deepening the meaning of their cultural heritage (Phalet & Baysu, 2020).

Friendships, especially when ethnically and culturally diverse, can provide opportunities to endorse both acculturation orientations (Vietze et al., 2019). In fact, experiences with same-ethnic friends are principally expected to orient ethnic minority adolescents to maintain their cultural heritage or to integrate the cultural heritage with the destination culture, whereas experiences with ethnic majority peers could play a role in transmitting the destination culture more so than the heritage culture (Motti-Stefanidi et al., 2012). In a nutshell, both in schools and friendships, ethnic minority adolescents can, according to their experiences, embrace cultural heritage maintenance and destination culture adoption.

### The Associations of Personal Identity Processes and Social Identifications with Acculturation Orientations in Education and Friendship Domains

Personal identity processes can be more intensive for ethnic minority youth because their minority status in the destination society implies that they need to negotiate different, and often opposing, identity alternatives proposed by heritage and destination cultures (Erentaitė et al., 2018). On the one hand, ethnic minority adolescents might be more uncertain about their identity choices and keep considering and reconsidering their commitments (Crocetti et al., 2011) as a result of the difficulties they encounter in balancing the customs and traditions of each culture. On the other hand, forming a coherent sense of self based on firm commitments may anchor minority youth to integrate the elements of both heritage and destination cultures (Schwartz et al., 2006, 2013). Thus, from a theoretical perspective, personal identity processes and acculturation orientations can be reciprocally linked.

So far, empirical evidence supporting this contention is still limited. A study conducted in Greece found that searching for and learning more about the meaning of ethnic background was found to decrease in-depth exploration (Mastrotheodoros et al., 2021). Another study conducted in the U.S. indicated bidirectional associations between personal identity coherence and sense of belongingness to heritage and destination societies (Meca et al., 2017). If this evidence suggests a possible interplay between identity and acculturation, accounting for how it unfolds in different domains can provide a more nuanced understanding of this phenomenon.

Indeed, youth might have less flexibility in exploring alternative identity possibilities and their preference for different acculturation orientations in education than in friendships. In schools, adolescents’ identity choices and acculturation orientations are constrained to a certain extent by educational settings (Becht et al., 2016; Klimstra et al., 2010) and by institutional values and policies around cultural diversity implemented by teachers (Phalet & Baysu, 2020). For instance, for adolescents to select a new school track (e.g., a vocational-oriented program) by changing the previous one, albeit not impossible, is rather complex. On the contrary, although friendships might be constrained by social homogamy, within these boundaries friendships have relatively limited constraints. For example, adolescents have relatively more freedom in choosing their friends and, if disappointed by their friendships, they can more easily give up on them and strive to establish new relationships. Thus, adolescents can explore and choose their commitments and acculturation orientations with more degrees of freedom in the friendship than in the education domain and, as a result, a stronger interplay between identity processes and acculturation orientations can be detected in the friendship domain.

In addition to personal identity processes, it is of utmost importance to consider the interplay of social identifications with classmates and the group of friends with acculturation orientations. Taking into account that engaging in harmonious interactions with classmates could enhance the sense of belongingness to the majority culture (Agirdag et al., 2011), social identification with classmates might be conceived as the main gateway to developing a sense of belongingness to the national group (Karataş et al., 2023). In this vein, greater social identification with classmates might be linked over time to adopting the destination culture more so than maintaining the heritage culture. Differently from social identification with classmates, higher identification with the group of friends might be conducive to endorsing both heritage and destination cultures because the group of friends is often composed of mutually chosen and potentially more diverse peers, which enables youth to engage in more frequent and intensive socialization experiences towards both cultures (Vietze et al., 2019; Wang et al., 2015). Therefore, while social identification with classmates might be associated predominantly with the adoption of the destination cultural elements in education, social identification with the group of friends may be linked to both acculturation orientations in the friendship domain.

## The Current Study

Ethnic minority youth cope with identity and acculturation tasks. The way in which they address them is likely to be intertwined but this is still poorly understood. The current study aimed to advance the understanding of this key contemporary societal issue in two main directions. First, it sought to consider how personal identity processes (i.e., commitment, in-depth exploration, and reconsideration of commitment) and social identifications are related over time to acculturation orientations (i.e., cultural heritage maintenance and destination culture adoption) by taking a domain-specific approach in which the two core life domains of adolescence (i.e., education and friendship) were considered. Second, in order to provide a more complete overview of this developmental interplay, associations were modeled considering whether effects could be mainly explained at a group (considering how adolescents’ variations from the group mean score on one variable are related to changes observed on another variable over time) or individual (examining how deviations from an adolescent’s own average score on one variable are related to changes in another variable over time) level. Thus, considering both levels can provide a more nuanced understanding of how identity processes, social identifications, and acculturation orientations are developmentally related in ethnic minority youth.

## Method

### Participants

Participants in this study were drawn from a larger longitudinal research project, Developing Inclusive Identities in Adolescence. Adolescents attending seven different high schools (i.e., lyceum, technical, and vocational high schools) located in small (about 25,000 inhabitants), medium (about 97,000 inhabitants), and large (about 150,000 inhabitants) cities in the North-East of Italy agreed to participate in this study at three different time points with six months in-between. Adolescents were in their first year of secondary high school at T1 and in their second year at T2 and T3.

The final longitudinal sample for the current study included 244 out of the 364 originally participating ethnic minority adolescents (56.6% female; *M*_age_ = 14.90, *SD*_age_ = 0.84, age range: 14 − 17 years at T1) who enrolled at least in two (out of three) time points of the data collection. As detailed in Supplementary Information, adolescents in the final longitudinal sample did not significantly differ from the overall sample across time on most of the demographic and study variables. All participants in the final longitudinal sample were recruited from multi-ethnic classrooms. The average percentage of ethnic minority adolescents in these classes was 27.4%.

The majority of participants (74.6%) were second-generation immigrants (born in Italy), whereas the remaining adolescents were first-generation immigrants who had been living in Italy for an average of 7.53 years (*SD* = 5.11; range: 6 months−15.5 years) at T1. Ethnic minority adolescents were fluent in Italian (*M* = 9.10, *SD* = 1.55, range: 0 − 10) and the fluency of second-generation immigrants (*M* = 9.44, *SD* = 0.83) was significantly (*t*(63.406) = − 4.189, *p* < 0.001) higher than that of first-generation ones (*M* = 8.07, *SD* = 2.50). Only six participants (2.5% of the sample) reported their language fluency to be lower than 5. During the questionnaire administration, they could ask for translation or clarification to trained research assistants or, in some cases, to other ethnic minority students from the same country.

Among the first-generation migrants, 67.7% were born in other European countries, with Romanians, Ukrainians, and Albanians as the most highly represented groups. The rest of the first-generation migrants were born in Africa (17.7%), Asia (8.1%), and North, Central, and South America (6.5%). Among second-generation immigrant adolescents, most parents migrated from other European countries (40.6 and 49.5% of fathers and mothers, respectively), with Albania being the most frequent. The remaining parents migrated from Africa (20.6 and 19.2% of fathers and mothers, respectively); Asia (2.8 and 2.7% of fathers and mothers, respectively); North, Central, and South America (3.9% of fathers, 6.6% of mothers); and the Middle East (1.1% of fathers, 0.5% of the mothers). Overall, these numbers reflect the socio-demographic characteristics of the ethnic minorities living in the Italian context. Both at the national (ISTAT, 2020) and local (Regione Emilia-Romagna, 2022) contexts, most immigrants living in Italy are from Eastern European countries, such as Romania (21.5% at the national and 17.5% at the regional levels) and Albania (8.3 and 10.5% at the national and local levels, respectively), followed by those of African (e.g., Moroccans, who represent the 8.3 and 11% of ethnic minorities at the national and regional levels, respectively) and Asian (e.g., Chinese, who represent almost 6.0% of the ethnic minority population at both levels) origins. Regarding the reasons for migration, the majority of participants reported that their parents had migrated to improve their family’s economic situation (35.2 and 29.5% of fathers and mothers, respectively), for family reunification (7.8 and 23.4% of fathers and mothers, respectively), other reasons (e.g., to study, to escape war; 3.6 and 6.9% of fathers and mothers, respectively), or did not answer this question (53.4 and 40.2% of fathers and mothers, respectively).

Concerning family structure, most participants (77.5%) reported that they came from two-parent families, 20.1% indicated that their parents were separated or divorced, and the others (2.4%) specified other family situations (e.g., one deceased parent). Fathers’ educational level was as follows: 45.7% held less than a high school diploma, 41.1% held a high school diploma, and 13.2% held a university degree. Mothers’ educational level was as follows: 30% held less than a high school diploma, 48.9% held a high school diploma, and 21.1% held a university degree.

To examine the distribution of missing values in the dataset, Little’s (1988) Missing Completely at Random (MCAR) was used. The findings showed a normed χ^2^ (χ^2^/*df*) of 0.98, *p* = 0.842, indicating that data were very likely missing at random. Thus, all participants in the final longitudinal sample (*N* = 244) were included in the analyses, and missing data were handled by means of the Full Information Maximum Likelihood (FIML; Kelloway, 2015) estimator in M*plus* 8.6 (Muthén & Muthén, 1998–2017).

### Procedure

This study was approved by the Ethics Committee of the Alma Mater Studiorum University of Bologna (Italy). Permission from school principals was obtained so that it could be possible to administer the study questionnaire during regular class hours at each time point. After obtaining permission from principals, students were provided with oral and written information about the study and were asked to sign informed consent forms. Besides active youth assent, active parental consent was also obtained by sending the parental consent forms to both parents at least one week before the date of the data collection at T1. Both youth assent and parental consent have been obtained from almost all (96.6%) of the approached ethnic minority and majority students and their parents in the Developing Inclusive Identities in Adolescence project.

At each time point, the school principals informed teachers (through written and digital communications) about the project and the scheduled data collection time. The teachers could stay in or leave the classroom during the questionnaire administration, whereas students without their assent or parental consent stayed in the classroom and did other school activities instead. The data collections at T1 (May 2019) and T2 (November 2019) were completed through a paper-and-pencil questionnaire administered in the classrooms, whereas the data collection at T3 (May 2020) was completed via an online version of the similar questionnaire due to the COVID-19 pandemic.^1^ Participants completed the questionnaire during school hours in about 30–40 min at each time point. For each participant a unique code was generated through which responses could be associated across the three time points while ensuring privacy. Participation in this longitudinal study was voluntary, and students were able to choose not to complete the questionnaire at each time point.

### Measures

Participants completed a questionnaire in Italian, including socio-demographic questions (e.g., age, biological sex, country of birth, time in Italy), the measures of personal identity processes, social identifications, and acculturation orientations.

#### Personal Identity

Commitment, in-depth exploration, and reconsideration of commitment were assessed using the Utrecht-Management of Identity Commitments Scale (U-MICS; Crocetti et al., 2008; for the Italian version, see Crocetti et al., 2010). The instrument consists of 13 items scored on a 5-point Likert-type rating scale, ranging from 1 (*completely untrue*) to 5 (*completely true*). All items were repeated twice to measure identity processes in education and friendship domains, separately. Sample items include: “My education/relation with my best friend gives me certainty in life” (commitment; 5 items), “I think a lot about my education/relation with my best friend” (in-depth exploration; 5 items), and “I often think it would be better to try to find a new education/best friend” (reconsideration of commitment; 3 items). Cronbach’s alphas for the three subscales ranged from 0.71 to 0.88 at T1; 0.76 to 0.87 at T2; 0.76 to 0.89 at T3 for the education domain, and from 0.71 to 0.87 at T1; 0.76 to 0.86 at T2; 0.76 to 0.88 at T3 for the friendship domain.

***Social Identifications***. Social identifications were measured with the Group Identification Scale (for both English and Italian versions, see Thomas et al., 2017). This measure consists of six items scored on a 5-point Likert-type scale, ranging between 1 (*completely false*) to 5 (*completely true*). Each item was presented twice to measure social identifications with classmates and the group of friends.^2^ A sample item is: “Belonging to the group of my classmates/friends is very important for who I am”. Cronbach’s alphas for social identifications with classmates and the group of friends ranged from 0.83 to 0.89 across time. Bivariate correlations between social identifications with these groups were 0.49, 0.52, and 0.34, respectively, at T1, T2, and T3, indicating a moderate overlap between the two groups across time.

#### Acculturation Orientations

Acculturation orientations were assessed with the Acculturation Strategies and Attitudes Scale (Navas et al., 2005; for the Italian version, see Mancini & Bottura, 2014). Cultural heritage maintenance (“How much do you currently maintain the traditions of your country of origin in each of the following domains [i.e., school, friendships]?”) and destination culture adoption (“How much have you adopted the traditions of the destination country (i.e., Italy) in each of the following domains [i.e., school, friendships]?”) were assessed with two items each (one for each domain). Items were scored on a 5-point Likert-type rating scale, ranging from 1 (*not at all*) to 5 (*very much*).

### Strategy of Analysis

As a preliminary step, descriptive statistics and bivariate correlations were estimated using the maximum likelihood estimator with robust standard errors (i.e., MLR; Satorra & Bentler, 2001) in M*plus*. Then, longitudinal measurement invariance for the domain-specific measures of personal identity processes and social identifications was tested. Next, both traditional and random-intercept cross-lagged panel models (Hamaker et al., 2015) were tested to examine associations of personal identity processes and social identifications with acculturation orientations, separately for the education and friendship domains. In each domain-specific model, an unconstrained model (M1) was initially estimated to identify the cross-lagged paths by controlling for stability paths (T1 → T2 and T2 → T3) and within-time correlations among all study variables at T1 and correlated changes at T2 and T3. To establish the model as parsimonious as possible, alternative models (M2) with cross-lagged paths constrained to be equal across time (i.e., T1 → T2 cross-lagged paths fixed to be equal to T2 → T3 paths) were estimated and compared to the baseline one (M1). Afterward, models (M3) in which both cross-lagged paths and correlated changes at T2 and T3 were constrained to be equal were tested and compared with the previous model (M2). Given that participants were nested within classrooms, standard errors were adjusted in each model by indicating the classroom as the cluster variable via the “*type=complex*” command available in M*plus*. Finally, as ancillary sensitivity analyses, the same models were tested by accounting for participants’ sex, age, immigrant status (i.e., first vs. second generation immigrant), time in Italy, and percentage of ethnic diversity in the classroom (in the educational model) and in the group of friends (in the friendship model) as the covariates.

To evaluate the model results, multiple criteria were considered: The Comparative Fit Index (CFI) with values higher than 0.90 representing an acceptable fit and values higher than 0.95 displaying an excellent fit; the Standardized Root Mean Square Residual (SRMR) and the Root Mean Square Error of Approximation (RMSEA), with values less than 0.08 indicative of an acceptable fit and values less than 0.05 indicating excellent fit (Byrne, 2012); and 90% Confidence Interval for the RMSEA, with the upper bound lower than 0.10 representing an acceptable model fit (Chen et al., 2008). To compare the nested models, the Satorra-Bentler (2001) scaled chi-square difference test and the changes (Δ) in the estimates of the CFI and RMSEA (Cheung & Rensvold, 2002) were evaluated. Models were considered different when at least two of the following criteria were matched: Δχ^2^_SB_ significant at *p* < 0.05 (Satorra & Bentler, 2001), ΔCFI ≥ −0.010, and ΔRMSEA ≥ 0.015 (Chen, 2007).

## Results

### Preliminary Analyses

Means and standard deviations are reported in Supplementary Table S1. Correlations are reported in Supplementary Table S2. Finally, as presented in Supplementary Table S3, the results of the longitudinal measurement invariance tests showed that full scalar invariance could be established for the measures of personal identity processes and social identifications.

### Domain-Specific Traditional Cross-Lagged Panel Models

As reported in Supplementary Table S4, for the education domain, the model comparison tests indicated that time invariance could be fully established for cross-lagged paths (M2) and correlated changes (M3). For the friendship domain, time invariance could be fully established for cross-lagged paths (M2) and partially for correlated changes (M3a). Accordingly, the more parsimonious models (M3 and M3a) for the education (*χ*^2^ = 113.934, *df* = 81, CFI = 0.955, SRMR = 0.062, RMSEA [90% CI] = 0.041 [0.021, 0.057]) and the friendship (*χ*^2^ = 133.735, *df* = 79, CFI = 0.933, SRMR = 0.058, RMSEA = 0.053 [0.037, 0.069]) domains were retained as the final ones. Standardized results of the domain-specific cross-lagged models are reported in Table 1.

**Table 1:** Standardized results of the traditional cross-lagged models

Stability paths	Education Domain		Friendship Domain^A^	
	T1 → T2	T2 → T3	T1 → T2	T2 → T3
Commitment	0.530^***^	0.522^***^	0.412^***^	0.323^***^
In-depth exploration	0.341^***^	0.285^***^	0.462^***^	0.412^***^
Reconsideration of commitment	0.544^***^	0.565^***^	0.371^***^	0.355^***^
Social identification^1^	0.545^***^	0.551^***^	0.520^***^	0.451^***^
Cultural heritage maintenance	0.483^***^	0.391^***^	0.375^***^	0.399^***^
Destination culture adoption	0.310^***^	0.210^*^	0.274^***^	0.198^*^
**Cross-lagged paths**	**T1** → **T2**	**T2** → **T3**	**T1** → **T2**	**T2** → **T3**
Commitment → In-depth exploration	0.232^***^	0.276^***^	0.011	0.012
Commitment → Reconsideration of commitment	0.012	0.014	−0.065	−0.071
Commitment → Social identification	0.185^**^	0.193^**^	0.111	0.122
Commitment → Cultural heritage maintenance	0.063	0.067	−0.025	−0.029
Commitment → Destination culture adoption	−0.028	−0.025	−0.027	−0.027
In-depth exploration → Commitment	0.012	0.013	0.077	0.080
In-depth exploration → Reconsideration of commitment	−0.011	−0.014	0.052	0.058
In-depth exploration → Social identification	−0.130^**^	−0.149^**^	0.025	0.027
In-depth exploration → Cultural heritage maintenance	−0.065	−0.077	−0.020	−0.023
In-depth exploration → Destination culture adoption	0.067	0.065	0.144	0.147
Reconsideration of commitment → Commitment	−0.100	−0.106	−0.035	−0.035
Reconsideration of commitment → In-depth exploration	0.001	0.001	−0.003	−0.004
Reconsideration of commitment → Social identification	−0.030	−0.034	−0.038	−0.041
Reconsideration of commitment → Cultural heritage maintenance	0.002	0.002	0.063	0.071
Reconsideration of commitment → Destination culture adoption	−0.108	−0.104	−0.078	−0.077
Social identification → Commitment	−0.010	−0.009	0.210^***^	0.252^**^
Social identification → In-depth exploration	−0.162^**^	−0.182^**^	0.079	0.100
Social identification → Reconsideration of commitment	−0.074	−0.084	−0.093	−0.119
Social identification → Cultural heritage maintenance	0.093	0.095	0.192^**^	0.258^**^
Social identification → Destination culture adoption	0.041	0.035	0.139^*^	0.164^*^
Cultural heritage maintenance → Commitment	0.117^*^	0.118^*^	0.081^*^	0.084^*^
Cultural heritage maintenance → In-depth exploration	0.068	0.083	0.073^*^	0.081^*^
Cultural heritage maintenance → Reconsideration of commitment	−0.048	−0.059	0.058	0.064
Cultural heritage maintenance → Social identification	0.038	0.041	0.024	0.027
Cultural heritage maintenance → Destination culture adoption	0.044	0.041	−0.016	−0.016
Destination culture adoption → Commitment	−0.020	−0.021	−0.030	−0.031
Destination culture adoption → In-depth exploration	−0.057	−0.072	0.029	0.031
Destination culture adoption → Reconsideration of commitment	−0.010	−0.013	−0.055	−0.060
Destination culture adoption → Social identification	−0.018	−0.020	−0.001	−0.001
Destination culture adoption → Cultural heritage maintenance	−0.131^*^	−0.150^*^	−0.183^**^	−0.210^**^

Within-time correlations	T1	T2	T3	T1	T2	T3
Commitment ↔ In-depth exploration	0.549^***^	0.471^***^	0.541^***^	0.567^***^	0.499^***^	0.516^***^
Commitment ↔ Reconsideration of commitment	−0.127	−0.069	−0.082	−0.238^**^	−0.153^*^	−0.162^*^
Commitment ↔ Social identification	0.370^***^	0.268^***^	0.277^***^	0.493^***^	0.430^***^	0.438^***^
Commitment ↔ Cultural heritage maintenance	0.026	0.038	0.039	0.067	0.163^**^	−0.030
Commitment ↔ Destination culture adoption	0.221^**^	0.187^**^	0.158^**^	0.374^***^	0.175^**^	0.166^*^
In-depth exploration ↔ Reconsideration of commitment	0.046	0.057	0.083	0.064	0.044	0.049
In-depth exploration ↔ Social identification	0.209^**^	0.254^***^	0.315^***^	0.346^***^	0.327^***^	0.352^***^
In-depth exploration ↔ Cultural heritage maintenance	0.163^*^	0.037	0.045	−0.008	0.023	0.028
In-depth exploration ↔ Destination culture adoption	0.233^***^	0.116	0.118	0.295^***^	0.075	0.076
Reconsideration of commitment ↔ Social identification	−0.019	0.061	0.079	−0.192^**^	−0.100	−0.111
Reconsideration of commitment ↔ Cultural heritage maintenance	0.201^*^	0.079	0.100	0.117	0.051	0.063
Reconsideration of commitment ↔ Destination culture adoption	0.053	−0.115^*^	−0.122^*^	−0.080	0.080	−0.125^*^
Social identification ↔ Cultural heritage maintenance	0.028	0.207^***^	0.227^***^	−0.040	0.003	0.004
Social identification ↔ Destination culture adoption	0.246^***^	0.153^*^	0.140^*^	0.391^***^	0.257^***^	0.254^***^
Cultural heritage maintenance ↔ Destination culture adoption	−0.024	0.044	0.039	0.050	0.138^*^	0.154^*^

T = Time; ^1^Social identification refers to social identifications with classmates and the group of friends in education and friendship domains, respectively. ^A^ In this model, correlated changes between commitment and maintenance, as well as between reconsideration of commitment and adoption were unconstrained

^*^p < 0.05, ^**^p < 0.01, ^***^p < 0.001

#### Identity and Acculturation in the Education Domain

**Fig. 1:**
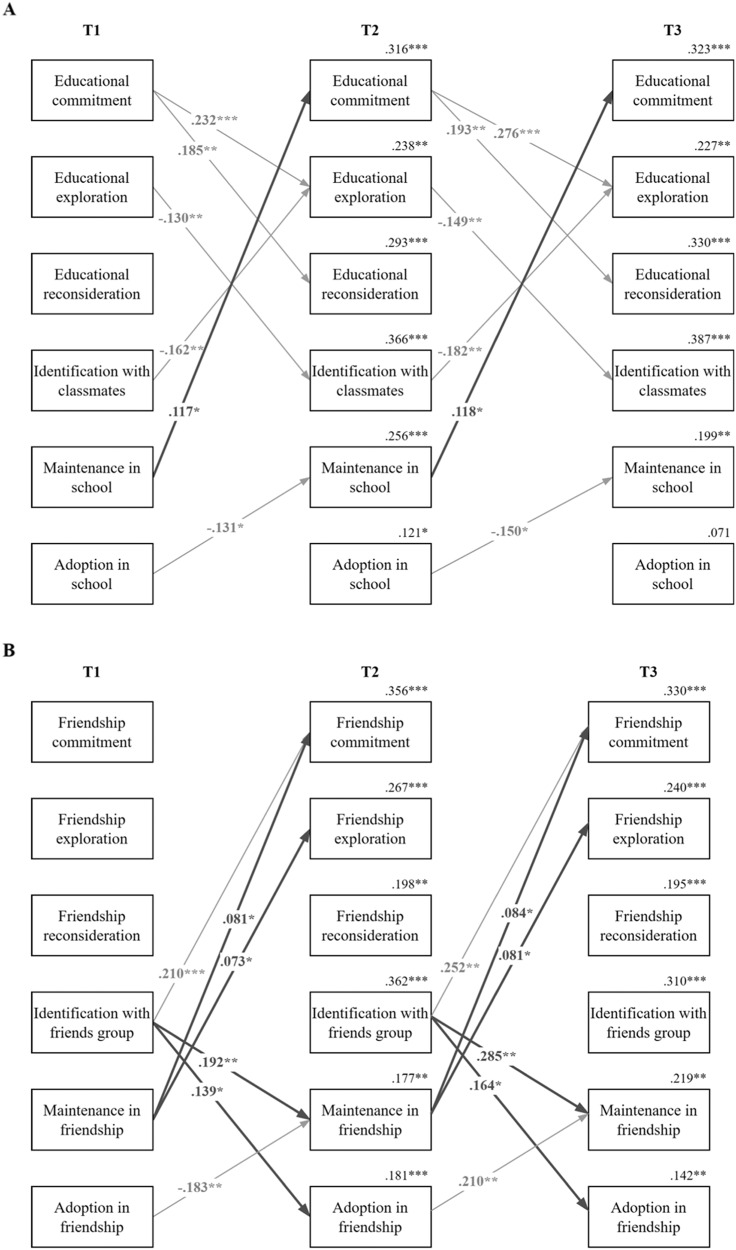
Significant Standardized Results of the Traditional Cross-lagged Model in Education (**A**) and Friendship Domains (**B**). Note. Bold arrows indicate the significant cross-construct associations while gray arrows indicate significant within-construct associations. T = Time. ^*^p < 0.05, ^**^p < 0.01, ^***^p < 0.001

The results highlighted one unidirectional cross-lagged effect from acculturation to identity processes in the educational domain. As depicted in Fig. 1A, adolescents who scored higher on cultural heritage maintenance compared to their peers, scored higher on commitment later on. As for the within-time correlations, educational identity commitment and social identification with classmates were positively correlated with destination culture adoption at T1, and these correlational patterns were also confirmed at T2 and T3. In-depth exploration was found to positively relate to both acculturation orientations at T1 but not at T2 and T3. Reconsideration of commitment was positively linked to cultural heritage maintenance only at T1, whereas it was negatively correlated with destination culture adoption at T2 and T3 but not at T1. Although the T1 correlations between social identification and cultural heritage maintenance were insignificant, correlated changes at T2 and T3 turned out to be positive and significant. Overall, these findings emphasized that identity processes and acculturation were somewhat interrelated.^3^

#### Identity and Acculturation in the Friendship Domain

The findings (see Fig. 1B) indicated unidirectional effects of acculturation on personal identity processes and unidirectional effects of social identification on acculturation. Specifically, adolescents who scored higher on cultural heritage maintenance compared to their peers, reported higher commitment and in-depth exploration later on, while those who scored higher on identification with friends reported over time also higher cultural heritage maintenance and destination culture adoption. Within-time correlations indicated that commitment and social identification with the group of friends were positively related to destination culture adoption at each time point, in-depth exploration was positively correlated with destination culture adoption only at T1, while changes in reconsideration of commitment were negatively associated with changes in destination culture adoption only at T3. Cultural heritage maintenance was positively and significantly correlated with commitment only at T2. The findings imply that personal identity processes and social identification with friends were moderately intertwined with the acculturation orientations in the friendship domain.

### Domain-Specific Random-Intercept Cross-Lagged Panel Models

As reported in Supplementary Table S5, for the education domain, the model comparison tests indicated that time invariance could be partially established for cross-lagged paths (M2a) and fully established for correlated changes (M3). For the friendship domain, time invariance could be fully established for cross-lagged paths (M2) and partially for correlated changes (M3a). Accordingly, the more parsimonious models (M3 and M3a) for the education (*χ*^2^ = 55.437, *df* = 56, CFI = 1.000, SRMR = 0.039, RMSEA = 0.000 [0.000, 0.039]) and the friendship (*χ*^2^ = 80.069, *df* = 58, CFI = 0.978, SRMR = 0.046, RMSEA = 0.039 [0.013, 0.059]) domains were retained as the final ones. Standardized results of the domain-specific random intercept cross-lagged models are reported in Table 2.^4^

**Table 2:** Standardized results of the random-intercept cross-lagged models

	Education Domain^a^		Friendship Domain^b^	
**WITHIN-PERSON EFFECTS**				
**Stability paths**	**T1** → **T2**	**T2** → **T3**	**T1** → **T2**	**T2** → **T3**
Commitment	0.138	0.292	0.510^**^	0.385
In-depth exploration	0.064	−0.079	−0.102	−0.311
Reconsideration of commitment	0.362^***^	0.307^*^	0.296	0.268
Social identification^1^	0.400	0.399	0.285	0.187
Cultural heritage maintenance	0.166	0.141	0.159	0.146
Destination culture adoption	−0.057	−0.130	0.047	−0.066
**Cross-lagged paths**	**T1** → **T2**	**T2** → **T3**	**T1** → **T2**	**T2** → **T3**
Commitment → In-depth exploration	0.060	0.077	0.283	0.328
Commitment → Reconsideration of commitment	0.003	0.004	0.109	0.124
Commitment → Social identification	0.125	0.137	0.112	0.131
Commitment → Cultural heritage maintenance	0.021	0.023	0.168	0.201
Commitment → Destination culture adoption	0.164	0.144	0.052	0.053
In-depth exploration → Commitment	−0.174	−0.184	−0.008	−0.009
In-depth exploration → Reconsideration of commitment	0.013	0.020	0.072	0.082
In-depth exploration → Social identification	−0.109	−0.140	0.001	0.002
In-depth exploration → Cultural heritage maintenance	−0.112	−0.141	−0.068	−0.081
In-depth exploration → Destination culture adoption	−0.119	−0.124	0.091	0.093
Reconsideration of commitment → Commitment	−0.121	−0.120	0.109	0.108
Reconsideration of commitment → In-depth exploration	0.050	0.070	0.120	0.130
Reconsideration of commitment → Social identification	0.018	0.021	0.001	0.001
Reconsideration of commitment → Cultural heritage maintenance	0.316^*^	−0.215	0.130	0.146
Reconsideration of commitment → Destination culture adoption	−0.088	−0.085	−0.071	−0.067
Social identification → Commitment	0.207	0.121	0.102	0.143
Social identification → In-depth exploration	−0.112	−0.144	0.021	0.032
Social identification → Reconsideration of commitment	−0.030	−0.040	−0.052	−0.078
Social identification → Cultural heritage maintenance	−0.008	−0.009	0.205	0.324
Social identification → Destination culture adoption	0.279	−0.017	0.087	0.117
Cultural heritage maintenance → Commitment	0.079	0.074	0.137	0.145
Cultural heritage maintenance → In-depth exploration	−0.027	−0.035	0.143	0.166
Cultural heritage maintenance → Reconsideration of commitment	−0.094	−0.128	0.079	0.090
Cultural heritage maintenance → Social identification	−0.096	−0.109	0.086	0.100
Cultural heritage maintenance → Destination culture adoption	0.038	0.129	−0.088	−0.089
Destination culture adoption → Commitment	0.018	0.019	−0.066	−0.070
Destination culture adoption → In-depth exploration	−0.166	−0.238	−0.062	−0.072
Destination culture adoption → Reconsideration of commitment	−0.012	−0.018	−0.092	−0.104
Destination culture adoption → Social identification	−0.029	−0.035	−0.024	−0.028
Destination culture adoption → Cultural heritage maintenance	−0.210	−0.252	−0.267^**^	−0.319^**^

Within-time correlations	T1	T2	T3	T1	T2	T3
Commitment ↔ In-depth exploration	0.374^***^	0.370^***^	0.457^***^	0.530^***^	0.569^***^	0.611^***^
Commitment ↔ Reconsideration of commitment	−0.018	−0.096	−0.117	0.098	−0.053	−0.054
Commitment ↔ Social identification	0.298	0.282^*^	0.288^*^	0.328	0.407^***^	0.428^***^
Commitment ↔ Cultural heritage maintenance	−0.165	−0.033	−0.032	0.276	0.258^*^	0.045
Commitment ↔ Destination culture adoption	0.323^*^	0.209	0.169	0.276	0.155^*^	0.144^*^
In-depth exploration ↔ Reconsideration of commitment	0.148	0.087	0.152	0.245	0.126	0.153
In-depth exploration ↔ Social identification	0.255	0.240^*^	0.348^*^	0.152	0.355^***^	0.438^***^
In-depth exploration ↔ Cultural heritage maintenance	0.132	−0.066	−0.092	0.114	0.079	0.108
In-depth exploration ↔ Destination culture adoption	0.115	−0.029	−0.033	0.168	0.061	0.066
Reconsideration of commitment ↔ Social identification	0.158	0.066	0.094	0.025	−0.086	−0.102
Reconsideration of commitment ↔ Cultural heritage maintenance	0.229	0.039	0.054	0.188	0.086	0.113
Reconsideration of commitment ↔ Destination culture adoption	0.144	−0.127	−0.144	−0.042	0.092	−0.183
Social identification ↔ Cultural heritage maintenance	−0.247	0.113	0.130	0.086	0.068	0.091
Social identification ↔ Destination culture adoption	0.248	0.142	0.134	0.241	0.242^**^	0.258^**^
Cultural heritage maintenance ↔ Destination culture adoption	−0.069	−0.048	−0.044	0.061	0.061	0.072

BETWEEN-PERSON EFFECTS		
**Correlations between Random Intercepts**		
Commitment ↔ In-depth exploration	0.815^***^	1.088
Commitment ↔ Reconsideration of commitment	−0.225	−4.512
Commitment ↔ Social identification	0.486	1.723
Commitment ↔ Cultural heritage maintenance	0.315	−1.911
Commitment ↔ Destination culture adoption	0.095	1.247
In-depth exploration ↔ Reconsideration of commitment	−0.088	−0.485
In-depth exploration ↔ Social identification	0.173	0.484^**^
In-depth exploration ↔ Cultural heritage maintenance	0.212	−0.242
In-depth exploration ↔ Destination culture adoption	0.408	0.468
Reconsideration of commitment ↔ Social identification	−0.307	−0.884
Reconsideration of commitment ↔ Cultural heritage maintenance	0.095	−0.498
Reconsideration of commitment ↔ Destination culture adoption	−0.151	−0.339
Social identification ↔ Cultural heritage maintenance	0.650	−0.301
Social identification ↔ Destination culture adoption	0.131	0.570^*^
Cultural heritage maintenance ↔ Destination culture adoption	0.045	0.128

T = Time; ^1^Social identification refers to social identifications with classmates and the group of friends in education and friendship domains, respectively

^a^In this model, regression paths from reconsideration to maintenance, from maintenance to adoption, from social identification to commitment, and from social identification to adoption were unconstrained

^b^In this model, correlated changes between commitment and maintenance, and between reconsideration and adoption were unconstrained

^*^p < 0.05, ^**^p < 0.01, ^***^p < 0.001

#### Identity and Acculturation in the Education Domain

**Fig. 2:**
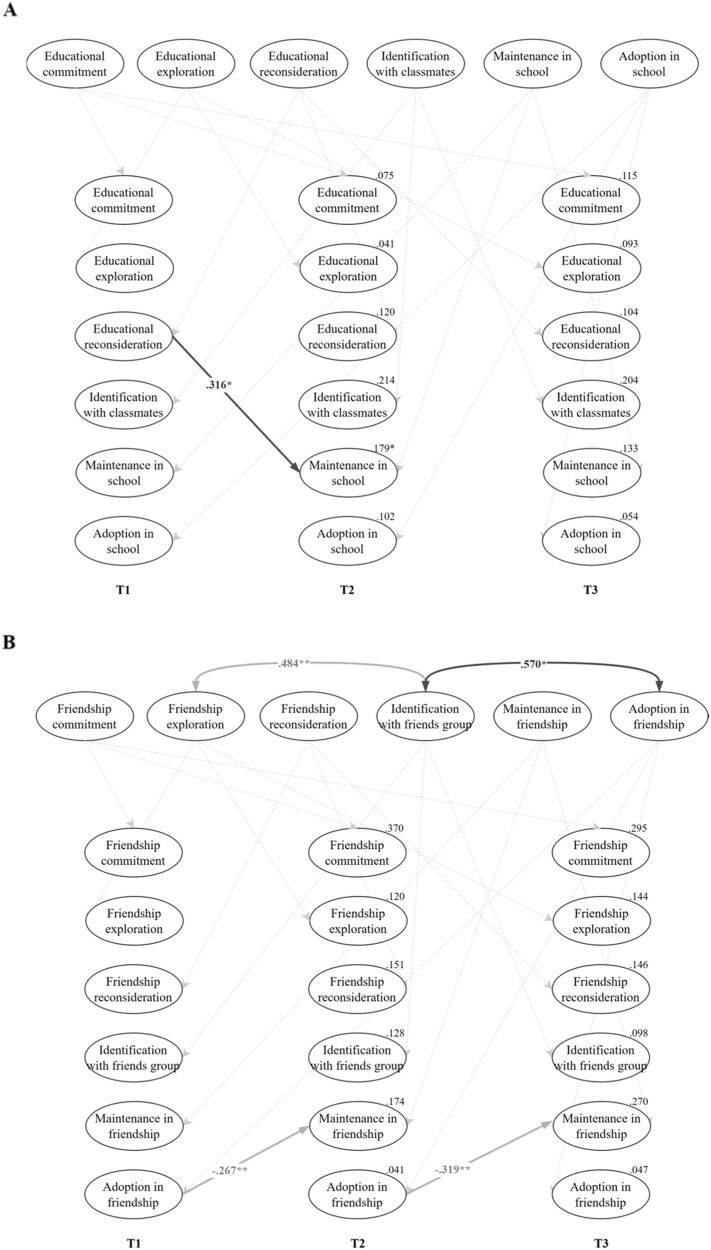
Significant Standardized Results of the Random-Intercept Cross-lagged Model in Education (**A**) and Friendship Domains (**B**). Note. Bold arrows indicate the significant cross-construct associations while gray arrows indicate significant within-construct associations. T = Time. ^*^p < 0.05, ^**^p < 0.01, ^***^p < 0.001

The results highlighted one unidirectional cross-lagged effect from identity processes in the educational domain to acculturation. As depicted in Fig. 2A, when adolescents reported above their own average on reconsideration of commitment, they reported increased cultural heritage maintenance later on. As for the within-time correlations, only one significant association emerged at the within-person level. Specifically, identity commitment correlated positively with destination culture adoption at T1 only. That is, when adolescents reported higher than average commitment in the education domain at T1, they also reported higher levels of destination culture adoption in the school context at that time. Overall, these findings only partially confirmed the intertwined nature of identity processes and acculturation at the within-person level.

#### Identity and Acculturation in the Friendship Domain

In the friendship domain, no significant cross-lagged effects emerged (see Fig. 2B).^5^ Within-time correlations indicated that changes in commitment and social identification with the group of friends were positively related to changes in acculturation orientations. Specifically, increases in levels of commitment were linked to increased heritage culture maintenance at T2 and to increased destination culture adoption at T2 and T3. Further, increases in social identification with the group of friends correlated with increases in destination culture adoption at T2 and T3. The findings imply that personal identity processes and social identification with friends were moderately intertwined with acculturation orientations in the friendship domain.

## Discussion

Adolescence is a pivotal period for the development of personal identity and social identifications (Crocetti et al., 2023). Fulfillment of these tasks may be more demanding for ethnic minority youth due to ongoing acculturation. The current study addressed the dynamic interplay between identity processes and acculturation in the education and friendship domains at the group and individual levels. The findings highlighted that most effects were at the group level. In the educational domain, adolescents who scored higher on cultural heritage maintenance compared to their peers, scored higher on commitment later on. In the friendship domain, stronger associations were found, such that adolescents who scored higher on cultural heritage maintenance compared to their peers, reported higher commitment and in-depth exploration later on, while those who scored higher on identification with friends reported over time also higher cultural heritage maintenance and destination culture adoption. At the individual level, when adolescents reported above their own average on reconsideration of educational commitment, they also reported increased cultural heritage maintenance later on. These findings were complemented by meaningful correlations both at baseline and across time, detected in all models. Overall, the current study provides not only insights into how personal and social facets of adolescents’ identities are intertwined with acculturation orientations but also underlines the domain-specific nuances that might play a role in these associations. Increasing the understanding of this complex phenomenon can pave the way for developing interventions and actions to enhance social inclusivity in contemporary diverse societies (Agi & Rivas-Drake, 2022; Guan et al., 2022).

### The Role of Cultural Heritage Maintenance in Driving Personal Identity Processes

Results of cross-lagged panel models indicated the pivotal role played by cultural heritage maintenance. In both the education and friendship domains, adolescents who endorsed this acculturation orientation relatively more than their peers, reported higher identity commitments over time. Additionally, in the friendship domain, this positive effect was extended also to in-depth exploration. Thus, maintaining cultural heritage elements in education and friendships was found to be a key asset for consolidating identity commitments in corresponding domains. These results can be interpreted in line with the risk and resilience model (Suárez-Orozco et al., 2018), according to which the fulfillment of acculturation tasks may function as an essential source for ethnic minority youth to meet the developmental task of forming a personal identity.

These findings, showing that cultural heritage maintenance in the education and friendship domains is linked to relative increases in making firm personal identity commitments in corresponding domains, have the potential to contribute integrative efforts combining personal identity processes and acculturation (e.g., Schwartz et al., 2013). When adolescents maintain values, traditions, and behaviors stemming from cultural heritage in terms of their ways of studying and living in the school institution, they can consolidate their identity choices in the education domain. Similarly, maintaining cultural heritage elements in choosing friends and the ways of spending time with them can foster interpersonal commitment. Because endorsement of cultural heritage elements can be driven by the acculturation expectations of the majority group members (e.g., Karataş et al., 2020; Vietze et al., 2020), providing schools and friends with less assimilationist majority expectations might be pivotal for youth developing a coherent sense of self (Phalet & Baysu, 2020). Thus, future research may uncover the roles of majority group members’ acculturation expectations in each life domain to unfold the underlying mechanism at play.

Another major finding is when adolescents reported higher cultural heritage maintenance in friendships compared to their peers, they also reported relatively higher in-depth exploration of commitment in the friendship domain over time. Such a finding not only confirms the role of cultural heritage maintenance in driving identity processes but also suggests that ethnic minority youth with stronger maintenance of cultural heritage may need to deepening and verifying the meaning of their identity commitments. So, embracing customs related to cultural heritage in friendships might make the friendships a secure arena (McLean & Jennings, 2012), facilitating the social processes of sharing personal experiences and feelings about already established commitments, as well as gathering feedback from others (Crocetti & Rubini, 2018). This study suggests that enacting such a social verification process in friendships is facilitated by cultural heritage maintenance.

### Social Identification with the Group of Friends as a Resource for Acculturation Orientations in the Friendship Domain

This study highlighted that adolescents who reported higher social identification with the group of friends compared to their peers, over time reported also higher endorsement of both acculturation orientations (i.e., heritage cultural maintenance and destination culture adoption). In the group of mutually chosen friends, ethnic minority adolescents can interact with both same-ethnic and cross-ethnic friends. Thus, they can experience positive and intimate forms of intergroup contact (Titzmann et al., 2015) that, as this study suggests, may represent a secure basis for deepening the meaning of their cultural heritage while also becoming more familiar with the destination culture. Therefore, social identification with the group of friends could orient youth to embrace both cultures simultaneously.

### From the Group to the Individual Level

The findings discussed so far refer to cross-lagged effects found at in the traditional cross-lagged panel models and, thus, they provide mainly insights on what happens when adolescents deviate from the average of their group. Especially for social psychological processes, like the ones investigated in the current study, these effects are of utmost relevance (Orth et al., 2021). Nevertheless, complementing them with the examination of within-person effects allow for a more comprehensive understanding of the phenomenon under investigation (Negru-Subtirica et al., 2020).

In this study, the random-intercept cross-lagged panel models indicated that only one cross-lagged effect was found to be significant. In the education domain, when adolescents’ levels of reconsideration of commitment deviated from their own average, they also reported over time higher levels of cultural heritage maintenance. This result is of great interest and might suggest that when adolescents experience a dissatisfaction regarding their current educational choice they turn to the values, practices, and expectations of the family of origin for getting guidance and support. Furthermore, when adolescents increase their reconsideration of commitment they may signal a failure of the school system that, instead of embracing multiculturalism, might have communicated assimilationist ideologies (Phalet & Baysu, 2020). In this situation, ethnic minority adolescents may perceive they “don’t fit” well and thus, reconsidering their educational choices, they can search for other school contexts. In line with these considerations, it is of utmost importance to further investigate the role played by the cultural diversity climate that each school institution implements and how it is perceived by the students (Karataş, Eckstein et al., 2023; Schachner et al., 2016).

### Virtuous Alliances: Within-Time Associations Between Identity Processes, Social Identifications, and Acculturation Orientations

This study highlighted that, in addition to the cross-lagged effects discussed so far, also meaningful within-time correlations were detected, in both traditional and random-intercept cross-lagged panel models. The most consistent associations were the positive links between commitment and social identification on the one hand, and destination culture adoption on the other hand. In fact, in the traditional cross-lagged models, these associations were found in both domains and in terms of both concurrent associations and correlated changes. In the random-intercept cross-lagged models, albeit not always significant, these associations were still the most consistent ones.

These results have important theoretical implications, suggesting that the developmental (finding identity commitments and identifying with salient peer groups) and acculturation (especially for what concern getting more and more familiar with the culture of the destination society) tasks tend to go hand-by-hand in the adolescent phase (Mastrotheodoros et al., 2021). Additionally, this evidence has also relevant practical implications for youth positive development (Motti-Stefanidi, 2019). Identity commitments (e.g., Crocetti, 2018), social identifications (e.g., Jetten et al., 2012), and acculturation orientations (e.g., Berry, 2005), are cornerstones of individuals’ well-being. Thus, a virtuous circle in which they co-develop and reinforce each other, in a “good goes together with good” pattern (Meeus, 2016, p. 1978), could be further promoted and enhanced by means of tailored psychosocial interventions.

### Strengths, Limitations, and Future Directions

The present longitudinal study should be considered in light of its strengths and shortcomings, which suggest directions for future research. First, this study highlights the longitudinal associations of personal identity processes and social identifications with acculturation orientations in education and friendships domains as the most central domains in adolescents’ life. However, the processes in these two domains operate simultaneously and either strengthen or interfere with each other (Crocetti et al., 2012). Therefore, future longitudinal research might further uncover the interaction between these domain-specific processes.

Second, the current study measured to what extent youth endorse heritage and destination cultures by principally referring to the customs and traditions of each. However, acculturation is conceived as a broader multidimensional concept encompassing cultural practices (e.g., cultural customs and traditions), values (e.g., belief systems), and identifications (i.e., ethnic and national identities; Schwartz et al., 2010). Herein, future research should investigate how personal identity and social identification processes related to acculturation orientations by disentangling these specific components.

Third, this study tackled the domain-specific associations over one year, coinciding with the starting phase in secondary high schools in the Italian educational system. Nevertheless, all the processes under examination are conceived as lifelong endeavors (Lee et al., 2020; Schwartz et al., 2018). In this regard, it is pivotal to conduct further studies with multiple yearly assessments that capture the identity and acculturation experiences that span the critical transitions in life (e.g., from secondary high school to university).

Fourth, in this study, longitudinal associations were modeled using both traditional and random-intercept cross-lagged panel models (Hamaker et al., 2015). In this way, it was possible to gain a comprehensive understanding of different effects at the group and individual levels. Given the ongoing methodological debate regarding pros and cons of these different analytic approaches and alternative ones (e.g., Asendorpf, 2021; Lucas, 2023; Orth et al., 2021), it is of utmost importance to continue to reflect thoroughly on which models could provide a better estimation of real life developmental and social-psychological phenomena.

Finally, the current sample consisted of a relatively heterogeneous group of ethnic minority adolescents. They were mainly second-generation immigrants with diverse cultural backgrounds, representative of the general characteristics of the migrant population in the geographical area where this study has been conducted (Regione Emilia-Romagna, 2013). Thus, future studies with more first-generation immigrant youth can expand the understanding of the domain-specific associations under examination.

## Conclusion

In contemporary diverse societies, adolescents strive to cope with identity and acculturation tasks. This is especially the case for ethnic minority youth, you may need to find their own way in the crossfire of the values and traditions of their family of origin and of the destination society. This longitudinal study highlights how fulfilling developmental and acculturation tasks relate to each other in core adolescents’ life domains. In the cross-lagged panel models, heritage cultural maintenance was found to be a key asset for committing in both education and friendship domains and for exploring in-depth interpersonal choices. These findings suggest that fulfilling acculturation tasks strengthens the formation of personal identity among ethnic minority youth. Furthermore, identification with friends was a resource for both maintaining the cultural heritage and adopting the destination culture. Plausibly, in the group of friends, ethnic minority adolescents can experiment in a safe laboratory diverse intergroup relationship and orient themselves accordingly. In the random-intercept cross-lagged panel models, higher reconsideration of educational commitment drove an increase in cultural heritage maintenance, perhaps as a way to turn to a safe base after experiencing dissatisfaction with the educational choice. Finally, in all models, consistent associations (at baseline and over time) emerged. Overall, this study points to virtuous alliances between the fulfillment of tasks related to adolescents’ development and acculturation.

### Supplementary information

Supplementary Information

## Footnotes

1 To collect data via online platforms, the necessary modifications in the previous ethical approval were also carried out, and the updated version of the ethical approval was obtained before initiating the data collection at T3.

2 Ethnic diversity in the group of friends was measured by a single item (“Thinking about your friends’ group, how many of them also belong to your parent’s group of origin?”) adapted from the perceived ingroup overlap measure of the social identity complexity (Roccas & Brewer, 2002; Brewer & Pierce, 2005). This item can be rated on a scale from 0 to 100%, and the mean was 25.5%.

3 As ancillary sensitivity analyses, each domain-specific model was estimated, including participants’ sex, age, immigrant generation, time in Italy, and ethnic diversity (in the classroom and friendship contexts for the respective models) as covariates. The results showed that model results were largely replicated after controlling for these covariates (see Table S7).

4 As ancillary sensitivity analyses, each domain-specific model was estimated, including participants’ sex, age, immigrant generation, time in Italy, and ethnic diversity (in the classroom and friendship contexts for the respective models) as covariates. The results showed that model results were largely replicated (see Table S8). However, it was not possible to conduct sensitivity analyses on the friendship model because of convergence issues.

5 Results of this model should be taken with strong caution because of warnings related to the fact that the latent variable covariance matrix is not positive definite.
