# Supplementary material for: Navigating Across Heritage and Destination Cultures: How Personal Identity and Social Identification Processes Relate to Domain-Specific Acculturation Orientations in Adolescence
Source: J Youth Adolesc. 2023 Sep 29;53(2):397–415. doi: 10.1007/s10964-023-01870-y (PMC10764387; doi:10.1007/s10964-023-01870-y)
Supplement: Supplementary file 1 — Supplementary Information [file 10964_2023_1870_MOESM1_ESM.docx]

**Supplementary Information for:**

Navigating Across Heritage and Destination Cultures: How Personal Identity and Social Identification Processes Relate to Domain-Specific Acculturation Orientations in Adolescence

**Sample Attrition Analyses**

A total of 1,295 ethnic majority (*n* = 931) and ethnic minority (*n* = 364) adolescents agreed to participate in the Developing Inclusive Identities in Adolescence Project. Of this total sample of adolescents, 710 participated in all three waves, 275 participated in two waves, and 310 participated only in one wave. The final longitudinal sample of the project has been generated by considering the participants who participated in at least two waves of data collection. As a result, this sample included 984 adolescents (76%) and comprised 740 ethnic majority and 244 ethnic minority adolescents.

To gather a better understanding of the sample attrition, we completed two sets of analyses. First, we initially compared the adolescents in the final longitudinal sample (*n* = 984) to those excluded from it (*n* = 311) in terms of the main study variables. Second, we focused on the ethnic minority subsample and compared ethnic minority adolescents in the final longitudinal sample (*n* = 244) with the ethnic minority adolescents excluded from it (*n* = 120).

**Final Longitudinal Sample of Ethnic Minority and Majority Adolescents versus Excluded Sample of Ethnic Minority and Majority Adolescents**

Initial analyses showed that ethnic minority adolescents were over- represented, whereas ethnic majority adolescents were under-represented in the excluded sample (*χ*^2^(1) = 22.233, *p* = .000, Cramer’s *V* = .131). As for the demographic variables, analyses indicated the over-representation of males (*χ*^2^(1) = 61.461, *p* = .000, Cramer’s *V* = .218), and adolescents from vocational high schools, compared to students from lyceum (*χ*^2^(2) = 25.975, *p* = .000, Cramer’s *V* = .142), in the excluded sample. Apart from these findings, the over-representation of older participants (*F* = 8.575, *p =* .003, η^2^ = .007, Cohen’s *d* = .189), participants with divorced parents (*χ*^2^(3) = 15.847, *p* = .001, Cramer’s *V* = .111), and mothers with less than a high school diploma (*χ*^2^(2) = 8.850, *p* = .014, Cramer’s *V* = .085) were also observed among the participants in the excluded sample.

Regarding study variables at T1, adolescents in the final longitudinal sample displayed higher levels of commitment (*F* = 31.754, *p =* .000, η^2^ = .030, Cohen’s *d* = 0.458; *F* = 7.472, *p =* .006, η^2^ = .007, Cohen’s *d* = 0.229 for education and friendship domains, respectively) and in-depth exploration in both domains (*F* = 13.062, *p =* .000, η^2^ = .013, Cohen’s *d* = 0.304; *F* = 4.372, *p =* .037, η^2^ = .004, Cohen’s *d* = 0.161, for education and friendship domains, respectively), as well as lower levels of reconsideration of commitment in the education domain (*F* = 9.952, *p =* .002, η^2^ = .010, Cohen’s *d* = 0.262), whereas reconsideration of commitment did not significantly differ in the friendship domain (*p* > .05). Moreover, participants in the final longitudinal sample reported higher levels of social identification with classmates (*F* = 7.359, *p =* .007, η^2^ = .007, Cohen’s *d* = 0.228) but not social identification with the group of friends (*p* > .05).

At T2, participants in the final longitudinal sample reported lower levels of reconsideration of commitment in the education domain (*F* = 6.071, *p =* .014, η^2^ = .006, Cohen’s *d* = 0.308), whereas they reported higher levels commitment in the friendship domain (*F* = 4.280, *p =* .039, η^2^ = .004, Cohen’s *d* = 0.244). At T3, the only significant difference indicated that adolescents in the final longitudinal sample reported lower levels of reconsideration of commitment in the friendship domain (*F* = 5.950, *p =* .015, η^2^ = .007, Cohen’s *d* = 0.442). The remaining study variables did not significantly differ at T2 and T3 across groups (*p_s_* > .05). Even though these significant differences, predominantly at T1, the corresponding effect sizes were mainly small or moderate. Therefore, the final longitudinal sample of the Developing Inclusive Identities in Adolescence Project, composed of ethnic minority and majority adolescents, could represent the overall sample across time.

**Final Longitudinal Subsample of Ethnic Minority Adolescents versus Excluded Subsample of Ethnic Minority Adolescents**

A total of 364 ethnic minority adolescents had enrolled in the Developing Inclusive Identities in Adolescence Project. Among them, 148 participated in all three waves, 97 participated in two waves, and 119 participated only in one wave. As a result, 244 ethnic minority youth participated in at least two (out of three) waves of data collection and were included in the final longitudinal sample. They were compared with those excluded from it (*n* = 120) regarding the main study variables.

Initial analyses indicated that ethnic minority participants included and excluded from the final longitudinal sample did not significantly differ from each other in terms of school type, age, country of birth, family structures as well as parents’ nationalities and educational levels (*p_s_* > .05), with the exceptions for their biological sex (*χ*^2^(1) = 9.829, *p*< .010, Cramer’s *V* = .165) and generational status (*χ*^2^(1) = 5.672, *p*< .05, Cramer’s *V* = .125). Inspections of standardized residuals indicated that all of them were lower than |2|. Thus, the two subsamples were rather similar in terms of socio-demographic variables.

As far as the main study variables (i.e., acculturation orientations, personal identity processes, and social identifications) are concerned, the participants in the final longitudinal sample did not significantly differ (*p_s_* > .05) at each time point (i.e., T1, T2, and T3), except for destination culture adoption in schools at T1 and T2, as well as commitment and in-depth exploration in the educational domain at T1. In detail, participants in the final longitudinal sample reported significantly higher levels of destination culture adoption in schools at T1 and T2 (for T1, *F* = 6.780, *p* < .05, η^2^ = .029, Cohen’s *d* = 0.375; and for T2, *F* = 8.732, *p* < .01, η^2^ = .039, Cohen’s *d* = 0.619). Likewise, adolescents in the final longitudinal sample indicated significantly higher levels of commitment (*F* = 6.317, *p* < .05, η^2^ = .023, Cohen’s *d* = 0.344) and in-depth exploration (*F* = 8.465, *p* < .01, η^2^ = .031, Cohen’s *d* = 0.400) in the education domain at T1. Considering the relatively limited number of significant differences with small or medium effect sizes, it might be concluded that the ethnic minority adolescents in the final longitudinal sample of this study could represent the overall sample across time.

**Descriptive Statistics and Bivariate Correlations**

**Table S1**

*Means (M) and Standard Deviations (SD) of Study Variables*

|  | **T1** | | **T2** | | **T3** | |
| --- | --- | --- | --- | --- | --- | --- |
|  | ***M*** | ***SD*** | ***M*** | ***SD*** | ***M*** | ***SD*** |
| **Education Domain** |  |  |  |  |  |  |
| Commitment | 3.463 | 0.797 | 3.374 | 0.810 | 3.321 | 0.823 |
| In-depth exploration | 3.198 | 0.714 | 3.144 | 0.777 | 3.230 | 0.689 |
| Reconsideration of commitment | 2.807 | 0.979 | 2.839 | 1.048 | 2.999 | 0.894 |
| Social identification with classmates | 3.195 | 0.865 | 3.182 | 0.862 | 3.094 | 0.786 |
| Cultural heritage maintenance | 2.593 | 1.393 | 2.539 | 1.430 | 2.427 | 1.347 |
| Destination culture adoption | 3.994 | 0.921 | 3.928 | 0.991 | 3.691 | 1.102 |
| **Friendship Domain** |  |  |  |  |  |  |
| Commitment | 3.737 | 0.782 | 3.700 | 0.784 | 3.674 | 0.812 |
| In-depth exploration | 3.408 | 0.754 | 3.457 | 0.776 | 3.424 | 0.746 |
| Reconsideration of commitment | 1.896 | 0.993 | 1.952 | 0.979 | 1.995 | 0.954 |
| Social identification with the groups of friends | 3.835 | 0.669 | 3.810 | 0.785 | 3.783 | 0.772 |
| Cultural heritage maintenance | 2.862 | 1.404 | 2.760 | 1.441 | 2.538 | 1.319 |
| Destination culture adoption | 3.797 | 1.056 | 3.871 | 1.069 | 3.699 | 1.105 |

**Table S2**

*Descriptive Statistics and Bivariate Correlations among T1 study variables*

|  | 1. | 2. | 3. | 4. | 5. | 6. | 7. | 8. | 9. | 10. | 11. | 12. |
| --- | --- | --- | --- | --- | --- | --- | --- | --- | --- | --- | --- | --- |
| 1. Age |  | -.045 | .022 | -.220^**^ | -.004 | .136 | .016 | .072 | .160^*^ | -.019 | .015 | .059 |
| 2. Sex | -.042 |  | -.078 | .020 | -.008 | -.271^***^ | .091 | .328^***^ | -.070 | .036 | -.046 | -.032 |
| 3. Parents’ educational level | .005 | -.084 |  | -.071 | -.036 | -.161^*^ | .038 | -.032 | -.122 | .067 | -.062 | .027 |
| 4. Immigrant generation | -.219^**^ | .020 | -.047 |  | .732^***^ | -.039 | -.021 | .054 | -.038 | .054 | -.099 | .157^*^ |
| 5. Time in Italy (years) | .005 | .002 | .012 | .736^***^ |  | -.094 | .039 | .112 | -.091 | .165^*^ | -.135^*^ | .234^***^ |
| 6. Ethnic diversity in the classroom//friends group | .083 | -.140 | .095 | -.108 | -.118 |  | -.082 | -.081 | .176 | -.007 | .282^***^ | -.028 |
| 7. Commitment | .070 | -.176^**^ | -.020 | -.097 | -.117^*^ | -.066 |  | .553^***^ | -.239^**^ | .483^***^ | .068 | .346^***^ |
| 8. Exploration | .096 | -.025 | -.091 | -.069 | -.049 | -.100 | .547^***^ |  | .083 | .316^***^ | -.011 | .286^***^ |
| 9. Reconsideration | .076 | -.012 | .052 | -.120 | -.136 | .091 | -.118 | .052 |  | -.197^**^ | .111 | -.082 |
| 10. Social identification | -.025 | -.163^*^ | .013 | .041 | .009 | .029 | .366^***^ | .206^**^ | -.016 |  | -.040 | .371^***^ |
| 11. Cultural heritage maintenance | -.034 | -.005 | -.008 | -.107 | -.195^**^ | .190^*^ | .027 | .172^*^ | .195^*^ | .004 |  | .062 |
| 12. Destination culture adoption | .037 | .120 | .024 | .128 | .112 | -.244^***^ | .188^*^ | .225^***^ | .050 | .222^**^ | -.017 |  |

*Note.* Sex: 0 = Male, 1 = Female; Immigrant generation: 0 = First generation, 1 = Second generation; T = Time.

Correlations for the education domain are presented below the diagonal whereas bivariate correlations for the friendship domain are reported above the diagonal.
^*^ *p* < .05, ^**^ *p* < .01, ^**^ *p* < .001

**Longitudinal Measurement Invariance Tests**

**Table S3**

*Longitudinal Measurement Invariance Tests for Each Study Measure*^1^

|  | **Model fit indices** | | | | | **Model comparison** | | | | | |
| --- | --- | --- | --- | --- | --- | --- | --- | --- | --- | --- | --- |
|  | *χ*_SB_^2^ | *df* | CFI | SRMR | RMSEA [90% CI] | Models | Δχ_SB_^2^ | Δ*df* | *p* | ΔCFI | ΔRMSEA |
| **Education Domain** | | | | | |  |  |  |  |  |  |
| Personal identity processes | | | | | |  |  |  |  |  |  |
| M1. Configural model | 447.219 | 288 | .940 | .059 | .048 [.039, .056] |  |  |  |  |  |  |
| M2. Metric model | 462.075 | 300 | .939 | .064 | .047 [.038, .055] | M2-M1 | 14.413 | 12 | .275 | -.001 | -.001 |
| M3. Scalar model | 495.106 | 318 | .934 | .066 | .048 [.039, .056] | M3-M2 | 33.612 | 18 | .014 | -.005 | -.001 |
| Social identification with classmates | | | | |  |  |  |  |  |  |  |
| M1. Configural model | 154.647 | 114 | .977 | .044 | .038 [.021, .053] |  |  |  |  |  |  |
| M2. Metric model | 165.929 | 124 | .976 | .051 | .037 [.020, .051] | M2-M1 | 10.918 | 10 | .364 | -.001 | -.001 |
| M3. Scalar model | 184.737 | 136 | .972 | .052 | .038 [.023, .052] | M3-M2 | 19.116 | 12 | .086 | -.004 | -.001 |
| **Friendship Domain** | | | | | |  |  |  |  |  |  |
| Personal identity processes | | | | | |  |  |  |  |  |  |
| M1. Configural model | 533.852 | 288 | .915 | .069 | .059 [.051, .067] |  |  |  |  |  |  |
| M2. Metric model | 550.155 | 300 | .914 | .073 | .058 [.051, .066] | M2-M1 | 15.887 | 12 | .196 | -.001 | -.001 |
| M3. Scalar model | 567.904 | 318 | .914 | .073 | .057 [.049, .062] | M3-M2 | 15.935 | 18 | .597 | .000 | -.001 |
| Social identification with the group of friends | | | | | |  |  |  |  |  |  |
| M1. Configural model | 198.060 | 114 | .944 | .061 | .055 [.042, .068] |  |  |  |  |  |  |
| M2. Metric model | 214.889 | 124 | .939 | .078 | .055 [.042, .067] | M2-M1 | 16.747 | 10 | .080 | -.005 | .000 |
| M3. Scalar model | 233.235 | 136 | .935 | .084 | .054 [.042, .066] | M3-M2 | 17.957 | 12 | .117 | -.004 | -.001 |

*Note.* χ_SB_^2^ = Satorra-Bentler scaled chi-square; *df* = degrees of freedom; CFI = Comparative Fit Index; TLI = Tucker-Lewis Index; SRMR = Standardized Root Mean Square Residual; RMSEA [90% CI] = Root Mean Square Error of Approximation and 90% Confidence Interval; Δ = Change in the parameter. ^1^ To test the longitudinal measurement invariance for the measures of personal identity processes in education and friendship domains, as a baseline, nine latent variables (for each subscale per time point) composed of either single items (for reconsideration of commitment) or parcels (for commitment and in-depth exploration) were generated. Three latent variables using single items were used to test the longitudinal measurement invariance for the measures of social identification with classmates and the group of friends.

**Model Fit Indices and Model Comparisons**

**Table S4**

*Traditional Cross-Lagged Models: Model Fit Indices and Model Comparisons*

| **Models** | **Model fit indices** | | | | | **Model comparison** | | | | | |
| --- | --- | --- | --- | --- | --- | --- | --- | --- | --- | --- | --- |
|  | *χ*^2^ | *df* | CFI | SRMR | RMSEA [90% CI] | Models | Δχ_SB_^2^ | Δ*df* | *p* | ΔCFI | ΔRMSEA |
| **Education Domain** |  |  |  |  |  |  |  |  |  |  |  |
| M1: Baseline model | 65.140 | 36 | .960 | .045 | .058 [.034, .080] |  |  |  |  |  |  |
| M2: Model with time-invariance of cross-lagged paths | 104.903 | 66 | .947 | .061 | .049 [.030, .066] | M2-M1 | 40.250 | 30 | .100 | -.013 | -.009 |
| M3: Model with time-invariance of T2– T3 correlations | 113.934 | 81 | .955 | .062 | .041 [.021, .057] | M3-M2 | 11.264 | 15 | .734 | .008 | -.008 |
| **Friendship Domain** | | | | | | | | | | | |
| M1: Baseline model | 85.777 | 36 | .939 | .039 | .075 [.055, .096] |  |  |  |  |  |  |
| M2: Model with time-invariance of cross-lagged paths | 112.845 | 66 | .942 | .051 | .054 [.036, .071] | M2-M1 | 30.131 | 30 | .459 | .003 | -.021 |
| M3: Model with time-invariance of T2– T3 correlations | 142.480 | 81 | .924 | .059 | .056 [.040, .071] | M3-M2 | 29.102 | 15 | .016 | -.018 | .002 |
| M3a: Model with partial time invariance of T2– T3 correlations^1^ | 133.735 | 79 | .933 | .058 | .053 [.037, .069] | M3a-M2 | 21.103 | 13 | .071 | -.009 | -.001 |

*Note*. χ_SB_^2^ = Satorra-Bentler scaled chi-square; *df* = degrees of freedom; CFI = Comparative Fit Index; TLI = Tucker-Lewis Index; SRMR = Standardized Root Mean Square Residual; RMSEA [90% CI] = Root Mean Square Error of Approximation and 90% Confidence Interval; Δ = Change in the parameter. ^1^ In this model, the correlated changes between heritage culture maintenance and identity commitment, and those between destination culture adoption and reconsideration of commitment were released.

**Table S5**

*Random Intercept Cross-Lagged Models: Model Fit Indices and Model Comparisons*

| **Models** | **Model fit indices** | | | | | **Model comparison** | | | | | |
| --- | --- | --- | --- | --- | --- | --- | --- | --- | --- | --- | --- |
|  | *χ*^2^ | *df* | CFI | SRMR | RMSEA [90% CI] | Models | Δχ_SB_^2^ | Δ*df* | *p* | ΔCFI | ΔRMSEA |
| **Education Domain** |  |  |  |  |  |  |  |  |  |  |  |
| M1: Baseline model | 13.465 | 15 | 1.00 | .017 | .000 [.000, .055] |  |  |  |  |  |  |
| M2: Model with time-invariance of cross-lagged paths | 72.749 | 45 | .967 | .046 | .050 [.027, .071] | M2-M1 | 58.040 | 30 | .002 | -.033 | .050 |
| M2a: Model with partial time-invariance of cross-lagged paths^1^ | 42.680 | 41 | .998 | .037 | .013 [.000, .046] | M2a-M1 | 28.771 | 26 | .322 | -.002 | .013 |
| M3: Model with time-invariance of T2– T3 correlations | 55.437 | 56 | 1.00 | .039 | .000 [.000, .039] | M3-M2 | 13.218 | 15 | .585 | .002 | -.013 |
| **Friendship Domain** | | | | | | | | | | | |
| M1: Baseline model | 23.019 | 15 | .992 | .023 | .047 [.000, .083] |  |  |  |  |  |  |
| M2: Model with time-invariance of cross-lagged paths | 61.802 | 45 | .983 | .037 | .039 [.003, .062] | M2-M1 | 39.090 | 30 | .124 | -.009 | -.008 |
| M3: Model with time-invariance of T2– T3 correlations | 91.198 | 60 | .968 | .048 | .046 [.025, .065] | M3-M2 | 27.665 | 15 | .024 | -.015 | .007 |
| M3a: Model with partial time invariance of T2– T3 correlations^2^ | 80.069 | 58 | .978 | .046 | .039 [.013, .059] | M3a-M2 | 18.171 | 13 | .151 | -.005 | .000 |

*Note*. χ_SB_^2^ = Satorra-Bentler scaled chi-square; *df* = degrees of freedom; CFI = Comparative Fit Index; TLI = Tucker-Lewis Index; SRMR = Standardized Root Mean Square Residual; RMSEA [90% CI] = Root Mean Square Error of Approximation and 90% Confidence Interval; Δ = Change in the parameter. ^1^ In this model, the correlated changes between heritage culture maintenance and identity commitment, and those between destination culture adoption and reconsideration of commitment were released. ^1^ In this model, the regression paths from reconsideration of commitment to heritage culture maintenance, from heritage culture maintenance to destination culture adoption, from social identification to identity commitment, and from social identification to destination culture adoption were released. ^2^ In this model, the correlated changes between commitment and heritage culture maintenance, and reconsideration of commitment and destination culture adoption were released.

**Sensitivity Analyses**

As ancillary sensitivity analyses, the traditional and the random-intercept cross-lagged panel models, which were retained as the final ones, were again estimated controlling for adolescents’ sex (0 = male, 1 = female), age, generational status (0 = first generation, 1 = second generation), time in Italy, and ethnic diversity in the classroom and friends’ group. The effects of the covariates measured at T1 were included in each model to predict T1, T2, and T3 variables. These effects were not constrained to allow them to vary across time. Model fit indices (see Table S6) indicated that the models including covariates fitted the data well in all cases, with the exception of the random-intercept cross-lagged panel model in the friendship domain (for which convergence was not reached when adding the covariates).

The findings of sensitivity analyses of the traditional and the random intercept cross-lagged panel models are reported in Tables S7 and S8, respectively.

**Table S6**

*Model Fit Indices of the Traditional and Random-Intercept Cross-Lagged Panel Models*

| **Models** | **Model fit indices** | | | | |
| --- | --- | --- | --- | --- | --- |
|  | *χ*^2^ | *df* | CFI | SRMR | RMSEA [90% CI] |
| **Cross-Lagged Panel Models** | | | | | |
| Education Domain | 124.980 | 81 | .950 | .048 | .050 [.032, .066] |
| Friendship Domain | 133.873 | 79 | .938 | .053 | .067 [.047, .086] |
| **Random-Intercept**  **Cross-Lagged Panel Model** | | | | | |
| Education Domain | 61.773 | 56 | .993 | .034 | .022 [.000, .049] |
| Friendship Domain | No convergence | | | | |

*Note*. χ_SB_^2^ = Satorra-Bentler scaled chi-square; *df* = degrees of freedom; CFI = Comparative Fit Index; TLI = Tucker-Lewis Index; SRMR = Standardized Root Mean Square Residual; RMSEA [90% CI] = Root Mean Square Error of Approximation and 90% Confidence Interval.

**Table S7**

*Standardized Results of the Traditional Cross-Lagged Models with Covariates*

| **Stability paths** | **Education Domain** | | | | **Friendship Domain** | | | | |  |
| --- | --- | --- | --- | --- | --- | --- | --- | --- | --- | --- |
|  | **T1 → T2** | | **T2 → T3** | | | **T1 → T2** | | **T2 → T3** | | |
| Commitment | .540^***^ | | .542^***^ | | | .473^***^ | | .451^***^ | | |
| In-depth exploration | .359^***^ | | .336^***^ | | | .362^***^ | | .309^**^ | | |
| Reconsideration of commitment | .551^***^ | | .547^***^ | | | .376^***^ | | .323^***^ | | |
| Social identification^1^ | .545^***^ | | .566^***^ | | | .532^***^ | | .540^***^ | | |
| Cultural heritage maintenance | .453^***^ | | .357^***^ | | | .295^***^ | | .488^***^ | | |
| Destination culture adoption | .249^**^ | | .242^**^ | | | .263^**^ | | .236^**^ | | |
| **Cross-lagged paths** | **T1 → T2** | | **T2 → T3** | | | **T1 → T2** | | **T2 → T3** | | |
| Commitment → In-depth exploration | .234^***^ | | .275^***^ | | | .086 | | 088 | | |
| Commitment → Reconsideration of commitment | .002 | | .002 | | | -.014 | | -.015 | | |
| Commitment → Social identification^1^ | .179^**^ | | .177^**^ | | | .106 | | .110 | | |
| Commitment → Cultural heritage maintenance | .037 | | .039 | | | .020 | | .024 | | |
| Commitment → Destination culture adoption | -.024 | | -.021 | | | .031 | | .032 | | |
| In-depth exploration → Commitment | .035 | | .037 | | | .015 | | .015 | | |
| In-depth exploration → Reconsideration of commitment | -.004 | | -.006 | | | .056 | | .061 | | |
| In-depth exploration → Social identification^1^ | -.114^*^ | | -.122^*^ | | | .065 | | .065 | | |
| In-depth exploration → Cultural heritage maintenance | -.041 | | -.047 | | | -.036 | | -.041 | | |
| In-depth exploration → Destination culture adoption | .057 | | .056 | | | -.092 | | .093 | | |
| Reconsideration of commitment → Commitment | **-.113^*^** | | **-.118^*^** | | | -.039 | | -.039 | | |
| Reconsideration of commitment → In-depth exploration | .004 | | .006 | | | .001 | | .001 | | |
| Reconsideration of commitment → Social identification^1^ | -.030 | | -.032 | | | -.074 | | -.075 | | |
| Reconsideration of commitment → Cultural heritage maintenance | -.006 | | -.007 | | | **.147^**^** | | **.168^**^** | | |
| Reconsideration of commitment → Destination culture adoption | -.106 | | -.105 | | | .014 | | .014 | | |
| Social identification^1^ → Commitment | .033 | | .030 | | | .243^***^ | | .290^***^ | | |
| Social identification^1^ → In-depth exploration | -.121^*^ | | -.135^**^ | | | **.131^*^** | | **.155^*^** | | |
| Social identification^1^ → Reconsideration of commitment | -.074 | | -.084 | | | **-.215^***^** | | **-.284^**^** | | |
| Social identification^1^ → Cultural heritage maintenance | .087 | | .087 | | | .232^**^ | | .317^**^ | | |
| Social identification^1^ → Destination culture adoption | .078 | | .067 | | | .166^*^ | | .202^*^ | | |
| Cultural heritage maintenance → Commitment | .113^*^ | | .113^*^ | | | **.058** | | **.059** | | |
| Cultural heritage maintenance → In-depth exploration | .067 | | .082 | | | .094^*^ | | .094^*^ | | |
| Cultural heritage maintenance → Reconsideration of commitment | -.080 | | -.099 | | | .046 | | .052 | | |
| Cultural heritage maintenance → Social identification^1^ | .035 | | .036 | | | .035 | | .035 | | |
| Cultural heritage maintenance → Destination culture adoption | .035 | | .033 | | | -.008 | | -.008 | | |
| Destination culture adoption → Commitment | -.061 | | -.064 | | | -.028 | | -.029 | | |
| Destination culture adoption → In-depth exploration | -.075 | | -.096 | | | .026 | | .027 | | |
| Destination culture adoption → Reconsideration of commitment | .003 | | .003 | | | .008 | | .009 | | |
| Destination culture adoption → Social identification^1^ | -.013 | | -.014 | | | -.024 | | -.025 | | |
| Destination culture adoption → Cultural heritage maintenance | -.111^*^ | | -.128^*^ | | | -.187^*^ | | -.219^*^ | | |
| **Covariates** | **T1 → T1** | **T1 → T2** | | **T1 → T3** | **T1 → T1** | | **T1 → T2** | | **T1 → T3** |  |
| Sex → Commitment | -.165^*^ | .085 | | .105 | .126 | | .000 | | .045 |  |
| Sex → In-depth exploration | -.015 | .060 | | .076 | .361^***^ | | .086 | | .141 |  |
| Sex → Reconsideration of commitment | .020 | -.050 | | .004 | -.028 | | -.109 | | -.130 |  |
| Sex → Social identification^1^ | -.138 | .017 | | -.077 | .076 | | .041 | | -.063 |  |
| Sex → Cultural heritage maintenance | .006 | -.044 | | -.110 | .036 | | -.022 | | -.115 |  |
| Sex → Destination culture adoption | .095 | .088 | | .132 | .000 | | .034 | | .006 |  |
| Age → Commitment | .083 | -.106 | | .017 | .067 | | .000 | | -.033 |  |
| Age → In-depth exploration | .085 | -.141^**^ | | -.031 | .080 | | -.148^*^ | | -.033 |  |
| Age → Reconsideration of commitment | .084 | -.073 | | .025 | .131 | | -.095 | | .056 |  |
| Age → Social identification^1^ | -.011 | .032 | | .015 | .005 | | .008 | | -.067 |  |
| Age → Cultural heritage maintenance | -.049 | .088 | | -.075 | -.088 | | .050 | | -.038 |  |
| Age → Destination culture adoption | .069 | -.068 | | -.051 | .079 | | -.006 | | -.199^*^ |  |
| Generational Status → Commitment | .013 | -.058 | | .187 | -.050 | | -.018 | | .026 |  |
| Generational Status → In-depth exploration | -.013 | .073 | | .092 | -.012 | | -.129 | | .065 |  |
| Generational Status → Reconsideration of commitment | .009 | .093 | | -.005 | .189^*^ | | .012 | | -.013 |  |
| Generational Status → Social identification^1^ | .089 | .123 | | .029 | -.071 | | .010 | | -.025 |  |
| Generational Status → Cultural heritage maintenance | .069 | .141 | | .107 | .016 | | .058 | | .181 |  |
| Generational Status → Destination culture adoption | .124 | .204 | | -.140 | .003 | | .165 | | -.090 |  |
| Time in Italy → Commitment | -.133 | .062 | | -.106 | .134 | | .008 | | -.098 |  |
| Time in Italy → In-depth exploration | -.046 | -.048 | | -.066 | .109 | | .162^*^ | | .017 |  |
| Time in Italy → Reconsideration of commitment | -.124 | -.051 | | .017 | -.244^**^ | | .062 | | .010 |  |
| Time in Italy → Social identification^1^ | -.051 | .045 | | -.058 | .302^*^ | | .158 | | .144 |  |
| Time in Italy → Cultural heritage maintenance | -.227^*^ | -.122 | | -.159 | -.154 | | -.204^*^ | | -.150 |  |
| Time in Italy → Destination culture adoption | -.014 | -.003 | | .144 | .233^*^ | | -.004 | | -.046 |  |
| Ethnic diversity^2^ → Commitment | -.122 | .022 | | -.066 | -.065 | | .082 | | -.061 |  |
| Ethnic diversity^2^ → In-depth exploration | -.130 | .028 | | -.048 | -.010 | | .156^*^ | | -.154 |  |
| Ethnic diversity^2^ → Reconsideration of commitment | .078 | .083 | | .070 | .134 | | .033 | | -.083 |  |
| Ethnic diversity^2^ → Social identification^1^ | .013 | .072 | | .041 | .041 | | .160^*^ | | .005 |  |
| Ethnic diversity^2^ → Cultural heritage maintenance | .173 | .053 | | -.041 | .294^***^ | | .057 | | .075 |  |
| Ethnic diversity^2^ → Destination culture adoption | -.196^**^ | -.113 | | -.009 | -.028 | | -.036 | | -.023 |  |
| **Within-time correlations** | **T1** | **T2** | | **T3** | **T1** | | **T2** | | **T3** |  |
| Commitment ↔ In-depth exploration | .536^***^ | .384^***^ | | .467^***^ | .546^***^ | | .488^***^ | | .514^***^ |  |
| Commitment ↔ Reconsideration of commitment | **-.146^*^** | -.103 | | -.125 | -.234^*^ | | -.166^*^ | | -.197^*^ |  |
| Commitment ↔ Social identification | .367^***^ | .250^***^ | | .258^***^ | .492^***^ | | .356^***^ | | .382^***^ |  |
| Commitment ↔ Cultural heritage maintenance | .027 | .015 | | .016 | .129 | | .161^*^ | | -.002 |  |
| Commitment ↔ Destination culture adoption | .218^**^ | .150^*^ | | .132^†^ | .364^***^ | | **.084** | | **.089** |  |
| In-depth exploration ↔ Reconsideration of commitment | .041 | .029 | | .042 | .101 | | .044 | | .050 |  |
| In-depth exploration ↔ Social identification | .220^***^ | .216^***^ | | .265^***^ | .325^***^ | | .251^***^ | | .256^***^ |  |
| In-depth exploration ↔ Cultural heritage maintenance | .190^*^ | .025 | | .031 | .028 | | -.017 | | -.023 |  |
| In-depth exploration ↔ Destination culture adoption | .211^***^ | .074 | | .078 | .291^***^ | | .063 | | .064 |  |
| Reconsideration of commitment ↔ Social identification | -.012 | .054 | | .066 | -.196^*^ | | -.039 | | -.045 |  |
| Reconsideration of commitment ↔ Cultural heritage maintenance | .169^*^ | .065 | | .080 | .061 | | .001 | | .001 |  |
| Reconsideration of commitment ↔ Destination culture adoption | .081 | -.150^**^ | | -.157^**^ | -.046 | | .135 | | **.043** |  |
| Social identification ↔ Cultural heritage maintenance | .018 | .201^**^ | | .211^**^ | -.026 | | -.037 | | -.052 |  |
| Social identification ↔ Destination culture adoption | .280^***^ | **.158** | | **.140** | .386^***^ | | .238^**^ | | .247^**^ |  |
| Cultural heritage maintenance ↔ Destination culture adoption | .036 | .043 | | .038 | .127 | | **.045** | | **.062** |  |

*Note.* T = Time; ^1^Social identification refers to social identifications with classmates and the group of friends in education and friendship domains, respectively.

^2^Ethnic diversity refers to the percentage of ethnic minority classmates and the amount of ethnic minority peers in the friends’ group for the education and friendship models, respectively. Results highlighted in **blue** are those that in the main models were not statistically significant, while they turned out to be significant when accounting for covariates. Results highlighted in **grey** are those that in the main models were statistically significant, while they lost significance when accounting for covariates.

^*^ *p* < .05, ^**^ *p* < .01, ^***^ *p* < .001, ^†^ *p* = 0.05.

**Table S8**

*Standardized Results of the Random-Intercept Cross-Lagged Models with Covariates*

|  | **Education Domain** | | | |  |
| --- | --- | --- | --- | --- | --- |
| **WITHIN-PERSON EFFECTS** | | | | |  |
| **Stability paths** | **T1 → T2** | | **T2 → T3** | | |
| Commitment | -.065 | | .271 | | |
| In-depth exploration | .044 | | -.062 | | |
| Reconsideration of commitment | .364^***^ | | .285^*^ | | |
| Social identification with classmates | .276 | | .368 | | |
| Cultural heritage maintenance | .144 | | .009 | | |
| Destination culture adoption | -.070 | | -.077 | | |
| **Cross-lagged paths** | **T1 → T2** | | **T2 → T3** | | |
| Commitment → In-depth exploration | .044 | | .058 | | |
| Commitment → Reconsideration of commitment | .006 | | .007 | | |
| Commitment → Social identification with classmates | .164 | | .170 | | |
| Commitment → Cultural heritage maintenance | -.007 | | -.008 | | |
| Commitment → Destination culture adoption | .215 | | .191 | | |
| In-depth exploration → Commitment | -.170 | | -.174 | | |
| In-depth exploration → Reconsideration of commitment | .028 | | .041 | | |
| In-depth exploration → Social identification with classmates | -.091 | | -.103 | | |
| In-depth exploration → Cultural heritage maintenance | -.126 | | -.150 | | |
| In-depth exploration → Destination culture adoption | -.146 | | -.142 | | |
| Reconsideration of commitment → Commitment | -.139 | | -.174 | | |
| Reconsideration of commitment → In-depth exploration | .056 | | .081 | | |
| Reconsideration of commitment → Social identification with classmates | .033 | | 038 | | |
| Reconsideration of commitment → Cultural heritage maintenance | .288^†^ | | -.270 | | |
| Reconsideration of commitment → Destination culture adoption | -.080 | | -.078 | | |
| Social identification with classmates → Commitment | .328 | | -.072 | | |
| Social identification with classmates → In-depth exploration | -.061 | | **-.078** | | |
| Social identification with classmates → Reconsideration of commitment | -.017 | | -.022 | | |
| Social identification with classmates → Cultural heritage maintenance | .062 | | .065 | | |
| Social identification with classmates → Destination culture adoption | .331 | | .008 | | |
| Cultural heritage maintenance → Commitment | .059 | | .057 | | |
| Cultural heritage maintenance → In-depth exploration | -.025 | | -.034 | | |
| Cultural heritage maintenance → Reconsideration of commitment | -.142 | | -.194 | | |
| Cultural heritage maintenance → Social identification with classmates | -.069 | | -.074 | | |
| Cultural heritage maintenance → Destination culture adoption | .113 | | .025 | | |
| Destination culture adoption → Commitment | .019 | | .019 | | |
| Destination culture adoption → In-depth exploration | -.183 | | **-.262^*^** | | |
| Destination culture adoption → Reconsideration of commitment | .021 | | .030 | | |
| Destination culture adoption → Social identification with classmates | -.013 | | -.015 | | |
| Destination culture adoption → Cultural heritage maintenance | -.225 | | -.265^*^ | | |
| **Within-time correlations** | **T1** | **T2** | | **T3** |  |
| Commitment ↔ In-depth exploration | .286^*^ | .214^*^ | | .269^*^ |  |
| Commitment ↔ Reconsideration of commitment | .031 | -.062 | | -.074 |  |
| Commitment ↔ Social identification with classmates | .270 | .242 | | .227 |  |
| Commitment ↔ Cultural heritage maintenance | -.167 | -.041 | | -.042 |  |
| Commitment ↔ Destination culture adoption | .283^*^ | .174 | | .134 |  |
| In-depth exploration ↔ Reconsideration of commitment | .073 | .094 | | .179 |  |
| In-depth exploration ↔ Social identification with classmates | **.250^*^** | **.152** | | **.228** |  |
| In-depth exploration ↔ Cultural heritage maintenance | .228 | .030 | | .050 |  |
| In-depth exploration ↔ Destination culture adoption | .084 | -.123 | | -.239 |  |
| Reconsideration of commitment ↔ Social identification with classmates | .190 | .155 | | .220 |  |
| Reconsideration of commitment ↔ Cultural heritage maintenance | .177 | -.050 | | -.078 |  |
| Reconsideration of commitment ↔ Destination culture adoption | **.280^**^** | -.205 | | -.239 |  |
| Social identification with classmates ↔ Cultural heritage maintenance | -.148 | .213 | | .263 |  |
| Social identification with classmates ↔ Destination culture adoption | .179 | .205 | | .068 |  |
| Cultural heritage maintenance ↔ Destination culture adoption | -.009 | -.139 | | -.140 |  |
| **BETWEEN-PERSON EFFECTS** | | | | |  |
| **Correlations between Random Intercepts** | | | | |  |
| Commitment ↔ In-depth exploration | .797^***^ | | | |  |
| Commitment ↔ Reconsideration of commitment | -.323 | | | |  |
| Commitment ↔ Social identification with classmates | **.428** | | | |  |
| Commitment ↔ Cultural heritage maintenance | .275 | | | |  |
| Commitment ↔ Destination culture adoption | .024 | | | |  |
| In-depth exploration ↔ Reconsideration of commitment | -.143 | | | |  |
| In-depth exploration ↔ Social identification with classmates | .170 | | | |  |
| In-depth exploration ↔ Cultural heritage maintenance | .228 | | | |  |
| In-depth exploration ↔ Destination culture adoption | .408 | | | |  |
| Reconsideration of commitment ↔ Social identification | -.383 | | | |  |
| Reconsideration of commitment ↔ Cultural heritage maintenance | .092 | | | |  |
| Reconsideration of commitment ↔ Destination culture adoption | -.241 | | | |  |
| Social identification with classmates ↔ Cultural heritage maintenance | .458 | | | |  |
| Social identification with classmates ↔ Destination culture adoption | .140 | | | |  |
| Cultural heritage maintenance ↔ Destination culture adoption | .132 | | | |  |
| **Covariates** | **T1 → T1** | **T1 → T2** | | **T1 → T3** |  |
| Sex → Commitment | -.163^*^ | -.013 | | .078 |  |
| Sex → In-depth exploration | -.024 | .028 | | .072 |  |
| Sex → Reconsideration of commitment | .015 | -.027 | | .001 |  |
| Sex → Social identification with classmates | -.150^*^ | -.084 | | -.140^*^ |  |
| Sex → Cultural heritage maintenance | .012 | -.072 | | -.160^*^ |  |
| Sex → Destination culture adoption | .097 | .102 | | .166^*^ |  |
| Age → Commitment | .080 | -.099 | | -.011 |  |
| Age → In-depth exploration | .085 | -.099 | | -.060 |  |
| Age → Reconsideration of commitment | .075 | -.021 | | .001 |  |
| Age → Social identification with classmates | -.008 | .025 | | .043 |  |
| Age → Cultural heritage maintenance | -.053 | .059 | | -.027 |  |
| Age → Destination culture adoption | .080 | -.059 | | -.050 |  |
| Generational Status → Commitment | .018 | -.059 | | .202^†^ |  |
| Generational Status → In-depth exploration | -.010 | .059 | | .145 |  |
| Generational Status → Reconsideration of commitment | .010 | .089 | | -.014 |  |
| Generational Status → Social identification with classmates | .083 | .173^†^ | | .120 |  |
| Generational Status → Cultural heritage maintenance | .081 | .166 | | .172 |  |
| Generational Status → Destination culture adoption | .124 | .236 | | -.038 |  |
| Time in Italy → Commitment | -.142 | -.025 | | -.171^*^ |  |
| Time in Italy → In-depth exploration | -.053 | -.111 | | -.199 |  |
| Time in Italy → Reconsideration of commitment | -.108 | -.096 | | .022 |  |
| Time in Italy → Social identification with classmates | -.050 | -.006 | | -.079 |  |
| Time in Italy → Cultural heritage maintenance | -.227^*^ | -.227^*^ | | -.266^*^ |  |
| Time in Italy → Destination culture adoption | -.006 | -.002 | | .089 |  |
| Ethnic diversity in the classroom → Commitment | -.122 | -.025 | | -.060 |  |
| Ethnic diversity in the classroom → In-depth exploration | -.133 | -.024 | | -.028 |  |
| Ethnic diversity in the classroom → Reconsideration of commitment | .075 | .113^*^ | | .099 |  |
| Ethnic diversity in the classroom → Social identification with classmates | .008 | .076 | | .084 |  |
| Ethnic diversity in the classroom → Cultural heritage maintenance | .171 | .152^*^ | | .043 |  |
| Ethnic diversity in the classroom → Destination culture adoption | -.209^**^ | -.166^*^ | | -.043 |  |

*Note.* T = Time. Results highlighted in **blue** are those that in the main models were not statistically significant, while

they turned out to be significant when accounting for covariates. Results highlighted in **grey** are those that in the main

models were statistically significant, while they lost significance when accounting for covariates.

^*^ *p* < .05, ^**^ *p* < .01, ^***^ *p* < .001; ^†^ *p* = .050.
